# Plasmid DNA Delivery to Cancer Cells with Poly(L-lysine)-Based Copolymers Bearing Thermally Sensitive Segments: Balancing Polyplex Tightness, Transfection Efficiency, and Biocompatibility

**DOI:** 10.3390/pharmaceutics17081012

**Published:** 2025-08-02

**Authors:** Mustafa Kotmakci, Natalia Toncheva-Moncheva, Sahar Tarkavannezhad, Bilge Debelec Butuner, Ivaylo Dimitrov, Stanislav Rangelov

**Affiliations:** 1 Department of Pharmaceutical Biotechnology, Faculty of Pharmacy, Ege University, Ankara Street 172/98 Campus-Bornova, 35040 Izmir, Türkiye; shr.tarkavan@gmail.com (S.T.); bilge.debelec@ege.edu.tr (B.D.B.); 2 Institute of Polymers, Bulgarian Academy of Sciences, Akad. G. Bonchev St. 103-A, 1113 Sofia, Bulgaria; ntoncheva@polymer.bas.bg (N.T.-M.); dimitrov@polymer.bas.bg (I.D.)

**Keywords:** gene delivery, polyplexes, transfection, polyanionic stress, dye exclusion, light scattering

## Abstract

**Background/Objectives.** Efficient nucleic acid delivery into target cells remains a critical challenge in gene therapy. Due to its advantages in biocompatibility and safety, recent research has increasingly focused on non-viral gene delivery. **Methods**. A series of copolymers—synthesized by integrating thermally sensitive poly(N-isopropylacrylamide) (PNIPAm), hydrophilic poly(ethylene glycol) (PEG) grafts, and a polycationic poly(L-lysine) (PLL) block of varying lengths ((PNIPAm)_77_-*graft*-(PEG)_9_-*block*-(PLL)_z_, z = 10–65)—were investigated. Plasmid DNA complexation with the copolymers was achieved through temperature-modulated methods. The resulting polyplexes were characterized by evaluating complex strength, particle size, zeta potential, plasmid DNA loading capacity, resistance to anionic stress, stability in serum, and lysosomal membrane destabilization assay. The copolymers’ potential for plasmid DNA delivery was assessed through cytotoxicity and transfection studies in cancer cell lines. **Results.** Across all complexation methods, the copolymers effectively condensed plasmid DNA into stable polyplexes. Particle sizes (60–90 nm) ranged with no apparent correlation to copolymer type, complexation method, or N/P ratio, whereas zeta potentials (+10–+20 mV) and resistance to polyanionic stress were dependent on the PLL length and N/P ratio. Cytotoxicity analysis revealed a direct correlation between PLL chain length and cell viability, with all copolymers demonstrating minimal cytotoxicity at concentrations required for efficient transfection. PNL-20 ((PNIPAm)_77_-*graft*-(PEG)_9_-*block*-(PLL)_20_) exhibited the highest transfection efficiency among the tested formulations while maintaining low cytotoxicity. **Conclusions.** The study highlights the promising potential of (PNIPAm)_77_-*graft*-(PEG)_9_-*block*-(PLL)_z_ copolymers for effective plasmid DNA delivery to cancer cells. It reveals the importance of attaining the right balance between polyplex tightness and plasmid release to achieve improved biocompatibility and transfection efficiency.

## 1. Introduction

Genetic disorders, cancer, and many other human diseases are associated with genetic abnormalities triggered by deficient or irregular gene expression [1,2,3]. Consequently, gene therapy offers a promising approach to treating such diseases by repairing/replacing defective genes or inhibiting the abnormal gene expression through delivering various exogenous nucleic acids (DNA, mRNA, or siRNA) into affected cells [4,5]. However, due to nucleic acids’ inherent properties, such as their low stability in physiological fluids, susceptibility to degradation, and low cellular uptake, it is of great importance to develop safe and efficient gene delivery systems capable of overcoming various physiological barriers [6,7]. There are two main types of gene delivery systems: viral and non-viral vectors. Initially, the viral vectors were developed and extensively evaluated both in vitro and in vivo due to their high transfection efficiency [8,9]. Viruses are capable of penetrating biological membranes and delivering the genetic material to the targeted locations. However, the viral vectors face significant safety concerns associated with strong host immune responses and insertional mutations [10]. Additionally, they are characterized by high production costs. Alternatively, various non-viral gene delivery methods are explored [11]. They offer several advantages over viral systems, including improved biocompatibility, enhanced safety, reduced immunogenicity, and efficient nucleic acid condensation [12]. Non-viral gene delivery vectors include lipid nanocarriers, inorganic nanoparticles, and natural or synthetic polymer-based nanocarriers, as well as various hybrids, e.g., polymer–lipid nanoparticles [13]. Among them, cationic polymers such as polyethylenimine (PEI), poly(L-lysine) (PLL), and poly(2-dimethylaminoethyl methacrylate) are the most promising candidates due to their ability to tightly bind with nucleic acids through electrostatic interactions, thus spontaneously forming stable nanosized complexes (polyplexes) [14,15]. Furthermore, the positive surface charge of the polyplexes promotes cellular uptake of nucleic acids and their endosomal escape, leading to improved transfection efficiency [16,17]. Although PEI is considered the gold standard in non-viral gene delivery in terms of efficacy and there are a number of clinical trials using PEI-based DNA or RNA formulations, it falls short when it comes to the cytotoxicity pattern [18,19,20]. Moreover, the polyplexes formed from cationic homopolymers and DNA may rapidly bind to plasma proteins, leading to their clearance from blood circulation. One of the most extensively applied approaches in cationic polymer-based polyplex modification is the incorporation of hydrophilic flexible polymers such as poly(ethylene glycol) (PEG) in order to shield the positive surface charges, thus providing “stealth” properties that inhibit blood protein adsorption [21,22,23]. Although the majority of clinically approved or under evaluation nanocarriers contain PEG, there are several issues, such as the decrease in both cellular uptake and endosomal escape (“PEG dilemma”), that still need to be addressed [24,25]. The advances in controlled polymerization processes and modification techniques have enabled the preparation of various biocompatible and biodegradable cationic copolymer-based nanocarriers of nucleic acids with finely tuned composition, architecture, functionalities, and physicochemical properties [26,27]. Thus, an effective surface functionalization of the vectors with appropriate targeting ligands for enhanced cellular uptake was achieved [28]. Moreover, various linkers or polymer segments responsive to external stimuli (e.g., pH-, temperature-, enzyme-, or redox-sensitive) were incorporated into the carriers in order to improve the specificity of gene delivery and to reduce toxicity in the non-targeted tissues [29].

Plasmid DNA (pDNA) is a circular, double-stranded, extrachromosomal DNA molecule capable of autonomous replication and transcription within a host cell. pDNA serves as a versatile tool for delivering genetic material into cells to achieve therapeutic effects [30]. Diverse applications of pDNA in investigating a variety of therapeutic strategies include delivery of therapeutic protein genes [31,32,33] and the components of clustered regularly interspaced palindromic repeats-associated protein 9 (CRISPR/Cas9) system for therapeutic gene editing [34,35,36,37], achieving therapeutic RNA interference through plasmid-encoded short interfering RNAs (siRNAs) [38] or micro RNAs (miRNAs) [39], and notably, applications as prophylactic and therapeutic vaccines [40,41]. Physicochemical properties play a crucial role in its stability, interaction with biological systems, and ultimately, its effectiveness in gene delivery applications. The phosphate backbone of DNA carries a significant negative charge at physiological pH (−1 charge per nucleotide) [42,43]. Despite its versatility, delivering pDNA effectively faces significant hurdles, including rapid degradation by extracellular and intracellular nucleases [44,45]. Even upon reaching cells, the negatively charged pDNA struggles to cross the cell membrane and often becomes degraded in the endosomes [46], hindering cytoplasmic release and subsequent nuclear entry, especially in non-dividing cells.

The development and optimization of non-viral gene delivery systems are crucial for realizing the full potential of pDNA-based gene transfer in pharmaceutical and biomedical applications, as these systems offer a safer and more versatile alternative to viral vectors [47,48]. Ongoing research is focused on improving their transfection efficiency, cell-type specificity, and biocompatibility to enable effective gene therapies and advanced research applications.

A series of hybrid copolymers have previously been synthesized as gene delivery vectors [49]. The copolymers, denoted as (PNIPAm)_77_-*graft*-(PEG)_9_-*block*-(PLL)_z_, combine a thermally responsive poly(N-isopropylacrylamide) (PNIPAm) block, hydrophilic poly(ethylene glycol) (PEG) grafts, and a polycationic poly(L-lysine) (PLL) block of varying lengths. The copolymers’ ability to condense DNA into polyplexes, their colloidal stability, and cytotoxicity have been investigated. They formed well-defined nanoparticles upon abrupt heating above the transition temperature of PNIPAm. These nanoparticles effectively condensed DNA into polyplexes, which exhibited exceptional colloidal stability at room temperature. The size and zeta potential of the polyplexes were influenced by the length of the PLL block and the N/P ratio (the ratio of amine groups on the copolymer to phosphate groups on DNA). Importantly, the copolymers, particularly those with shorter PLL blocks and their corresponding polyplexes, showed minimal cytotoxicity. Therefore, it was concluded that these (PNIPAm-*graft*-PEG)-*block*-PLL copolymers hold promise as biocompatible gene delivery vectors, providing a solid foundation for the development of new and improved non-viral gene delivery systems [49]. Despite their promising performance, the capacity of these copolymers to mediate plasmid DNA delivery has not been evaluated. Reinforcing the relevance in further evaluating the potential applications of the (PNIPAm-*graft*-PEG)-*block*-PLL copolymers, in this contribution, we expand previous studies through further elaboration on the complexation of the copolymers with plasmid DNA, their physicochemical characterization, and their transfection efficiency on various cell lines.

## 2. Materials and Methods

### 2.1. Synthesis of Copolymers

To synthesize the hybrid copolymers (PNIPAm)_77_-*graft*-(PEG)_9_-*block*-PLL_z_, a three-step synthetic procedure was employed, as described in detail elsewhere [49]. Briefly, in the first step, the (PNIPAm)_77_-*graft*-(PEG)_9_ macroinitiator, bearing an ammonium hydrochloride terminal group, was obtained by free radical polymerization of N-isopropylacrylamide (NIPAm) and oligo(ethylene glycol) methacrylate (9 oxyethylene units, M_n_ = 500 g/mol) using potassium persulfate as the initiator and 2-aminoethanethiol hydrochloride as the chain transfer agent, via a grafting-through technique [49]. A detailed characterization of the (PNIPAm)_77_-*graft*-(PEG)_9_ block, including its thermo-responsive properties (lower critical solution temperature, LCST) is given in Table S1.

In the second step, the macroinitiator was used to initiate the ring-opening polymerization of the N-carboxyanhydride of Z-L-lysine (ZLLys-NCA) in DMF at 60 °C. In the third step, the Z-protecting groups were cleaved from the polypeptide chain, yielding a series of four hybrid copolymers (PNIPAm)_77_-*graft*-(PEG)_9_-*block*-PLL_z_, with the polypeptide block degree of polymerization (DP) ranging from 10 to 65 and a fixed (PNIPAm)_77_-*graft*-(PEG)_9_ block. The composition, molar mass characteristics, and code of the copolymers are given in Table 1. The synthetic route for the preparation of the copolymers is shown in Scheme S1.

### 2.2. Preparation of Polyplexes

The plasmid pEZX-MR04 (#CmiR0001-MR04) (Genecopoeia, Rockville, MD, USA) encoding enhanced green fluorescent protein (eGFP) was used as the model nucleic acid. The plasmid was amplified using standard molecular biology techniques. To test the complex formation between (PNIPAm)_77_-*graft*-(PEG)_9_-*block*-(PLL)_z_ copolymers and the pDNA, the different amine-to-phosphate ratios were tested. The complexation was tested under three different conditions: (1) The copolymer solution, properly diluted in HEPES-buffered glucose (HBG, 20 mM HEPES, and 5% glucose) was first heated to 65 °C for 5 min in the polypropylene micro centrifuge tubes. Then, the pDNA solution was added in an equal volume of HBG and mixed by pipetting. Immediately after mixing, the tube was brought to RT and incubated for 40 min. This method is referred to as Hot (H) complexation. (2) The copolymer solution was first heated to 65 °C for 5 min as described, then brought to RT for 5 min. The pDNA solution was added, mixed, and incubated at RT for 40 min. This method is referred to as Heat–cool (H-C) complexation. (3) Without applying any heating, the copolymer and the pDNA were mixed at RT and left for complexation at room temperature for 40 min. This method is referred to as room temperature (RT) complexation. All complexes were prepared in a final volume of 20 µL and the amount of the plasmid was kept constant at 200 ng per complex for all samples. For light scattering measurements, the dispersions were prepared in a final volume of 1 mL following the above protocols.

### 2.3. Agarose Gel Retardation

After the incubation, 4 µL of 6× DNA loading dye (30% glycerol *v*/*v*, 0.25% bromophenol blue *w*/*v*, and 0.25% xylene cyanol FF *w*/*v*) was added to the samples and mixed. For each, 20 µL of the mixture was loaded into the pockets of 1% agarose gel in 1× TAE buffer (40 mM Tris, 20 mM acetic acid, and 1 mM EDTA). An equal amount of naked pDNA in buffer was loaded to the gel as a reference. Electrophoresis was performed at 100 V for 20 min in a MyGel-Mini electrophoresis system (Accuris Instruments, Edison, NJ, USA). The pDNA bands were stained with 0.5 µg/mL ethidium bromide and the gels were visualized under UV illumination using the Syngene G:Box gel documentation system (Syngene, Cambridge, UK).

### 2.4. Ethidium Bromide Exclusion Assay

To test the capability of the copolymers to condense pDNA molecules into a compact form that prevents ethidium bromide intercalation, we performed an ethidium bromide (EtBr) exclusion assay as described previously, with modifications [50]. Briefly, the complexes were prepared as described in the previous section and 20 µL was aliquoted into the wells of a 96-well plate. Subsequently, 80 µL of 400 ng/mL ethidium bromide solution in HBG was added. After incubation for 10 min, the EtBr fluorescence was measured at λ_ex_/λ_em_ 510/590 nm. In preliminary experiments, it was observed that the copolymers are quenching nearly 25% of the EtBr fluorescence (Figure S1). For this reason, all measurements were performed with corresponding blanks that contained the copolymer and the EtBr solution at equivalent concentrations for background subtraction.

### 2.5. Polyanionic Stress Resistance

In this experiment, we used heparin, a high-charge density anionic macromolecule with high binding affinity to cationic copolymers [51]. Briefly, complexes were prepared as described above. After the complex formation, samples were transferred in 20 µL volumes into the wells of a 96-well plate and 400 ng/mL EtBr solution was added to each sample. After incubation for 10 min at RT, the fluorescence was measured at λ_ex_/λ_em_ 510/590 nm using a multi-well plate reader (Varioskan Flash, Thermo Fisher Scientific, Vantaa, FI, USA). A stock solution of heparin (50 IU/mL in HBG buffer consisting of 20 mM HEPES and 5% glucose, at pH 7.4) was added to the complexes by the automatic dispenser of the device. Starting with a dispensed volume of 2 µL per sample, the volume was increased by 1 µL after every fifth injection to achieve a relatively steady increase in heparin concentration. After each heparin addition, to achieve equilibrium in the samples, a shaking step and incubation for 5 min was performed before each fluorometric measurement. The temperature was kept constant at 37 °C throughout the experiment.

The equilibrium state of the pDNA (A) complexed by the copolymer (B) is represented by A:B ⇌ A + B, and the dissociation rate constant is presented by the following equilibrium expression:

*K_d AB_* = [*A*][*B*]/[*A*:*B*] (1)

The intercalation of ethidium bromide (C) with the pDNA is represented by A + C ⇌ A:C, and the association rate constant is presented by the following equilibrium expression:

*K_a AC_* = [*A:C*]/([*A*][*C*]) (2)

Assuming that the association of A and C takes place instantaneously right after the dissociation of A and B with the same rate, the effective dissociation constant can be found.

*K_d AB_* = *K_a AC_* = *K_d eff_* (3)

The fluorescence data were fitted to a binding isotherm using the Michaelis–Menten equation (Equation (4)):

[*A*:*C*] = ([*A*:*C*]*_max_* × [*D*])/(*K_d eff_* + [*D*]), (4)

where [A:C] represents the amount of free pDNA intercalated by ethidium bromide; [A:C]_max_ is the maximum observed amount of [A:C]; D is the concentration of heparin; and K_d eff_ is the effective dissociation constant that represents the concentration of heparin at which half of the A:B complex is dissociated. As the concentration of [A:B] is indirectly estimated by the fluorescence signal (F), Equation (4) can be rewritten as follows:

[*F*] = (*F_max_* × [*D*])/(*K_d eff_* + [*D*]) (5)

### 2.6. Serum Stability Test

Proteins present in serum are another stress factor that affects the polyplex stability both in vitro and in vivo [51]. In addition to the complex destabilization, serum nucleases lead to rapid degradation of free nucleic acids in cell culture media and in the blood circulation. To test the effect of serum proteins on the complex stability and the protection of the nucleic acid payload against nuclease degradation, complexes were incubated with FBS and subsequently analyzed by agarose gel electrophoresis. Briefly, after preparation, copolymer–pDNA complexes were mixed with non-inactivated fetal bovine serum (1:1 *v*/*v*) and incubated at 37 °C. The final FBS concentration was either 10% to represent the in vitro cell culture conditions, or 50% to represent the in vivo conditions. To test the decomplexation of pDNA by the presence of serum proteins, after the incubation, samples were directly loaded to 1% agarose gel and analyzed as described above.

To evaluate the protective effect of the copolymers against serum nucleases, after incubation with FBS, samples were first treated by a decomplexation buffer to liberate the pDNA, and its integrity was analyzed by agarose gel electrophoresis. Briefly, a decomplexation buffer was added in a 1:10 *v*/*v* ratio to the prepared complexes to provide 200 ng/mL of proteinase K, 0.5% of SDS, and 100 IU/mL of heparin in the final reaction. Samples were incubated at 56 °C for 40 min to activate proteinase K cleavage. Immediately after the incubation, samples were cooled on ice for 2 min, spun down, and processed for agarose gel electrophoresis as described above. Proteinase K was used in order to digest the serum proteins, and heparin was added in excess to enable decomplexation, whereas SDS served as an anionic detergent to induce destabilization of colloidal particles.

### 2.7. Physicochemical Characterization of the Polyplexes

#### 2.7.1. Electrophoretic Light Scattering (ELS)

ELS measurements were performed on a NanoBrook 90 Plus PALS instrument (Brookhaven Instruments, Nashua, NH, USA) equipped with a 35 mW solid-state laser, operating at λ = 660 nm. The principle of phase analysis light scattering (PALS) was applied for the measurements of the electrophoretic mobility. The ζ potential values were calculated from the obtained electrophoretic mobility using the Smoluchowski equation:

*ζ* = 4*πημ*/*ε*, (6)

where η is the solvent viscosity, υ is the electrophoretic mobility, and ε is the dielectric constant of the solvent. The final measurements are the average of the ten repeated ones, with an error smaller than ±3 mV.

#### 2.7.2. Static and Dynamic Light Scattering (SLS and DLS)

Light scattering measurements were carried out in the interval of angles from 40° to 140° using a Brookhaven BI-200 goniometer with vertically polarized incident light at a wavelength λ = 633 nm, supplied by a He–Ne laser operating at 35 mW and equipped with a Brookhaven BI-9000 AT digital autocorrelator. The scattered light was measured for dilute aqueous dispersions of the investigated polyplexes in the concentration range 0.024–0.735 mg/mL. The SLS data were analyzed using the Zimm plot software provided by Brookhaven Instruments. Information on the weight-average molar mass, M_w_, the radius of gyration, R_g_, and the second virial coefficient, A_2_, was obtained from the dependence of the quantity Kc/R_θ_ on the concentration (c) and scattering angle (θ). Here, K is the optical constant given by K = 4π^2^n_0_^2^(dn/dc)^2^/N_A_λ^4^, where n_0_ is the refractive index of the solvent (water), N_A_ is Avogadro’s constant, λ is the laser wavelength, R_θ_ is the Rayleigh ratio at θ, and dn/dc is the refractive index increment measured in water in separate experiments on an Orange GPC19 DNDC refractometer. Values of dn/dc in the 0.091–0.188 mL/g range depending on the copolymer composition and N/P ratio were found for the polyplexes.

The autocorrelation functions from DLS were analyzed using the constrained regularized algorithm CONTIN [52] to obtain the distributions of the relaxation rates (Γ). The latter provided distributions of the apparent diffusion coefficient (D = Γ/q^2^), where q is the magnitude of the scattering vector given by q = (4πn/λ)sin(θ/2), and n is the refractive index of the medium. The mean hydrodynamic radius was obtained by the Stokes–Einstein equation:

*R_h_* = *k_B_T*/(6*πηD*_0_), (7)

where k_B_ is the Boltzmann constant, η is the solvent viscosity at temperature T in Kelvin, and D_0_ is the diffusion coefficient at infinite dilution.

DLS measurements were carried out also, on a NanoBrook 90 Plus PALS instrument (Brookhaven Instruments), equipped with a 35 mW solid-state laser, operating at λ = 660 nm at a scattering angle of 90°. The samples were conditioned 10 min before each measurement. The hydrodynamic diameters (D_h_) were determined according to the Stokes–Einstein equation:

*D_h_* = *k_B_T*/(3*πηD*_90_), (8)

where D_90_ is the diffusion coefficient determined at an angle of 90°. Each measurement was performed in triplicate.

All dispersions were filtered through a 0.45 μm Nylon filter before measurements. For selected samples, measurements were performed at 37 °C. Since the differences in the values of light scattering parameters at 25 °C and 37 °C were within the standard deviations of the methods (0.5–6%), the results at 25 °C are presented.

#### 2.7.3. Atomic Force Microscopy (AFM)

AFM images were taken on a Bruker NanoScope V9 instrument with a 1.00 Hz scan rate under ambient conditions. For AFM sample preparation, a droplet of 2 μL dispersion was placed onto a freshly cleaned glass substrate (1 cm^2^) and spin-casted at 2000 rpm for 1 min. Observations were performed in ScanAsyst (Peak Force Tapping) mode.

### 2.8. Cell Culture

All cell lines used in this study were obtained from the American Type Culture Collection (ATCC). The following cell lines were included: PC3 (prostate cancer), H1299 and HCC827 (non-small cell lung cancer), SK-MEL-30 (melanoma), and HaCaT (human keratinocytes). PC3 cells were cultured in a 1:1 mixture of Dulbecco’s Modified Eagle Medium (DMEM) and Ham’s F-12 nutrient mix (DMEM/F-12), supplemented with 10% fetal bovine serum (FBS). SK-MEL-30 cells were cultured in DMEM/F-12 supplemented with 5% FBS. H1299 cells were cultured in RPMI-1640 medium supplemented with 10% FBS, and HCC827 and HaCaT cells were cultured in RPMI-1640 medium supplemented with 5% FBS. To prevent microbial contamination, all culture media were supplemented with 100 IU/mL penicillin and 100 μg/mL streptomycin. Cell cultures were maintained at 37 °C in a humidified atmosphere with 5% CO_2_. Cell growth and morphology were monitored every other day using an inverted light microscope. Upon reaching approximately 90% confluence, cells were detached using trypsin/EDTA and sub-cultured for experimental use and to maintain stock cultures.

### 2.9. Cytotoxicity Assay by Resazurin Cell Proliferation Test

To test the cytotoxicity of the copolymers on the cell lines, cells were seeded at a density of 5 × 10^3^ cells/well in a 96-well plate 24 h before the experiment. The copolymers were dissolved at a 10 mg/mL concentration in ultrapure water and kept in the fridge until use. On the day of treatment, initial dilution in full media was made to prepare a 1 mg/mL working solution. Subsequent serial dilutions were prepared in full media. The media of cells were aspirated and 100 µL fresh media containing the copolymers was added to the cells. Cells were incubated with copolymers at 37 °C in a humidified atmosphere with 5% CO_2_ for 48 and 72 h.

Cell viability was measured by the resazurin cell proliferation test. Resazurin is a blue dye which is internalized by live cells and metabolically reduced to form resorufin, a highly fluorescent pink compound. The amount of the resorufin formed is directly proportional to the metabolic activity in cells and its fluorescence intensity (FI) is used for estimating the viable cells as normalized to the untreated control [53]. Briefly, fresh media containing resazurin were prepared immediately before use by mixing five parts of the corresponding full medium with one part of 0.015% resazurin in PBS. The treatment media were carefully aspirated from the wells and 120 µL of the resazurin-containing media was added. After incubation at 37 °C for 3 h, the FI was measured using a multi-well plate reader (Varioskan Flash, Thermo Fisher Scientific, Vantaa, FI, USA). The FI of untreated cells was used for normalization of the intensity obtained from treated cells. A blank without cells, consisting of the corresponding medium and the resazurin, was used for background subtraction. The viability was calculated according to Equation (9):

*Viability* (%) = [(*FI_S_* − *FI_B_*)/(*FI_UT_* − *FI_B_*)] × 100, (9)

where FI_S_ is the fluorescence intensity of the treated sample, FI_B_ is the fluorescence intensity of the blank, and FI_UT_ is the fluorescence intensity of the untreated sample. All treatments were performed at least in triplicate and the results are expressed as mean ± SD. Non-linear regression analysis was used to calculate the IC_50_ values of the copolymers.

### 2.10. In Vitro Transfection Efficiency and Cytotoxicity of the Copolymer–pDNA Complexes by Flow Cytometry

Cells were seeded in 24-well plates at a density of 6 × 10^4^ cells/well in 500 µL of complete growth medium. The following day, at least 30 min prior to treatment, the culture medium was replaced with 450 µL of fresh complete growth medium. Complexes were prepared as described above. Cells were treated with the complexes at a pDNA concentration of 1 µg/mL. The treatment volume constituted 10% of the total culture medium volume for all experimental groups. HBG treatment served as a vehicle control. DharmaFECT 1 (Horizon Discovery, Lafayette, CO, USA) was used as a commercial transfection control according to the manufacturer’s protocol. Transfection was performed for 72 h. Following incubation, cells were washed twice with PBS and detached using 100 μL of 0.025% trypsin–EDTA for 5–7 min at 37 °C. Trypsinization was then quenched by adding complete medium, and the cells were transferred to microcentrifuge tubes. Cells were pelleted by centrifugation at 200× *g* for 5 min and resuspended in PBS containing 10% FBS for subsequent flow cytometry analysis. Transfection efficiency was analyzed using a BD Accuri C6 flow cytometer (BD Biosciences Piscataway, NJ, USA). Untreated cells were used to establish baseline fluorescence and define gates for subsequent analyses. For each sample, at least 10,000 events within a predetermined gate were recorded. Transfected cells were identified and quantified based on fluorescence detected with the FITC/GFP filter (FL-1).

### 2.11. Lysosomal Membrane Destabilization Assay

To test the ability of the copolymers to induce endosomal escape, an acridine orange (AO)-based lysosomal membrane destabilization assay was performed by modifying previously reported protocols [54,55]. Briefly, 8.5 × 10^3^ cells were seeded in 96-well plates in 100 µL of complete growth medium. The next day, the medium was refreshed, polyplexes at N/P 18 were prepared as described above, and cells were treated with the polyplexes at a pDNA concentration of 1 µg/mL. As a control, 50 μM chloroquine diphosphate (Cq) was used to induce lysosomal membrane destabilization. Cells were treated for 24 h and washed three times with 100 μL PBS (pH 7.4). Fresh medium containing 2 μg/mL AO was then added to cells and incubated at 37 °C for 15 min. Subsequently, cells were washed twice with 100 μL complete medium for 5 min at 37 °C, and once with phenol-free medium. After the washings, 50 μL of phenol-free medium was added to cells. Fluorescence measurement was performed using a Varioskan Flash multimode plate reader at λ_ex/em_ 465/650 nm. AO-treated cells were also imaged by a fluorescence microscope and representative images are presented after background subtraction using ImageJ software (version 1.52). To eliminate the impact of cytotoxicity due to treatments, a resazurin assay was performed on cells treated in the same way and the fluorescence data were normalized to the live cell fraction.

### 2.12. Statistical Analysis

All experiments were performed at least in triplicate, unless otherwise specified. The results are presented as mean ± SD or mean ± SEM. Multiple groups were analyzed by one-way analysis of variance and Tukey’s post-hoc test was performed to test the significance of the differences. Student’s *t* test was also used for single comparisons. In cases where *p* < 0.05, the difference between groups were considered significant.

## 3. Results and Discussion

### 3.1. Cytotoxicity of the Copolymers

**Figure 1:**
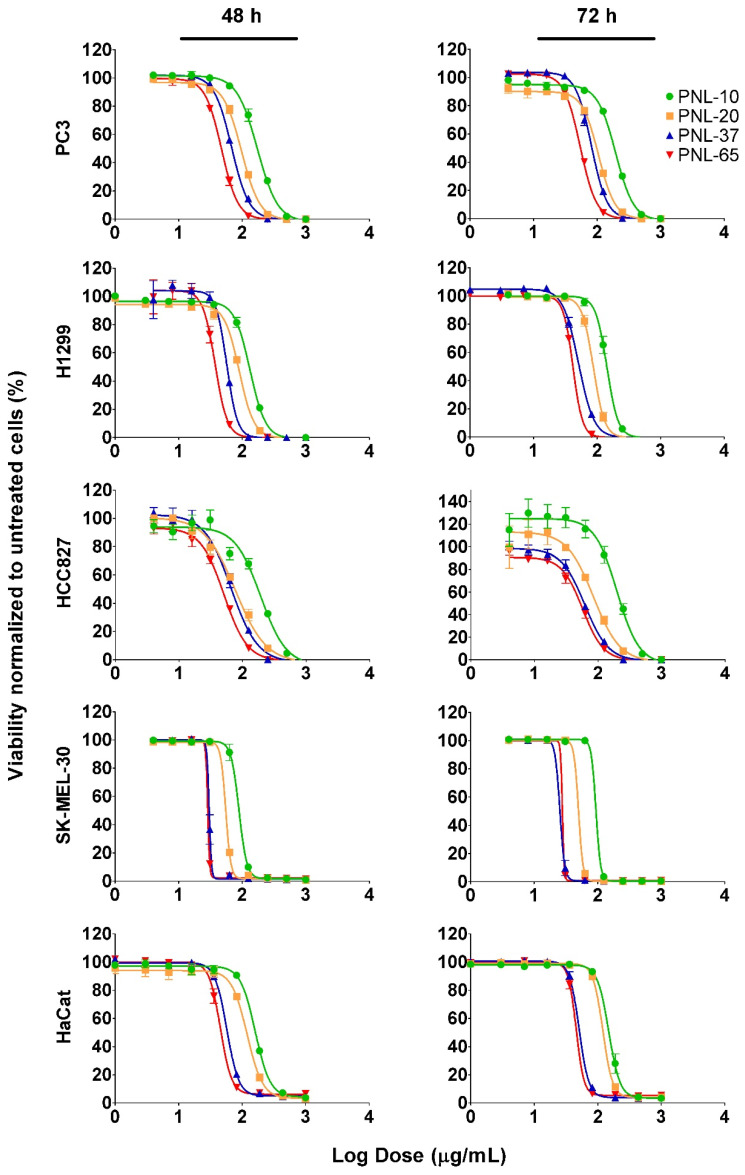
Cytotoxicity of the copolymers tested on PC3, H1299, HCC827, SK-MEL-30, and HaCaT cell lines for 48 and 72 h are shown. The copolymers’ codes and symbols are given in the upper-right-hand graphic.

Cytotoxicity of the copolymers was evaluated by treating PC3, H1299, HCC827, SK-MEL-30, and HaCaT cells with increasing copolymer concentrations for 48 and 72 h. Cell viability data are presented in Figure 1, and IC_50_ values are summarized in Table 2. PNL-10 exhibited the lowest toxicity, while PNL-65 was the most toxic, suggesting a direct correlation between poly(L-lysine) chain length and cytotoxicity. Notably, IC_50_ values were similar across most cell lines, with the exception of SK-MEL-30, indicating a potentially shared internalization mechanism. This consistency in cytotoxicity across diverse cell lines suggests a general mechanism of cytotoxicity for these copolymers.

### 3.2. Complexation of (PNIPAm)_77_-Graft-(PEG)_9_-Block-(PLL)_z_ Copolymers with pDNA

**Figure 2:**
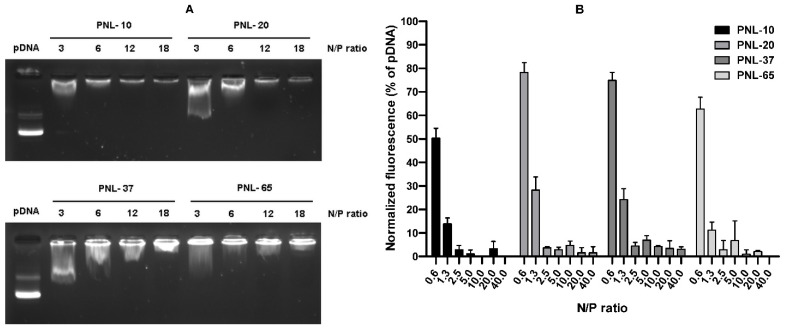
Complex formation ability of the copolymers with pDNA. (**A**) Agarose gel images showing the retardation of the pDNA bands by the copolymers at increasing N/P ratios, and (**B**) ethidium bromide exclusion assay results demonstrating the pDNA availability for ethidium bromide intercalation at different N/P ratios. Complexation was performed by simple mixing and incubating at RT for 45 min.

Complexation of the copolymers with pDNA has been tested with N/P ratios in the 0.5–40 range. The results showing retardation of pDNA due to complexation at an increasing N/P ratio on agarose gel electrophoresis are presented in Figure 2A. In addition, an EtBr exclusion assay was also utilized for semi-quantitative assessment of the pDNA compaction, and the results are presented as % EtBr fluorescence intensity normalized to pDNA alone. An N/P ratio-dependent decrease of EtBr fluorescence was detected at relatively low N/P ratios for complexes of all copolymers (Figure 2B). Thus, the increasing N/P ratio is suggested to result in higher compaction of the pDNA by all copolymers. A similar EtBr exclusion profile was previously reported for linear polyethylenimine and its histidinylated derivatives [56]. The plasmid compaction achieved by the preparation methods H-C and H was also evaluated. The resulting compaction patterns were similar across all complexation methods (Figure S2).

### 3.3. Physicochemical Characteristics of the Polyplexes

**Figure 3:**
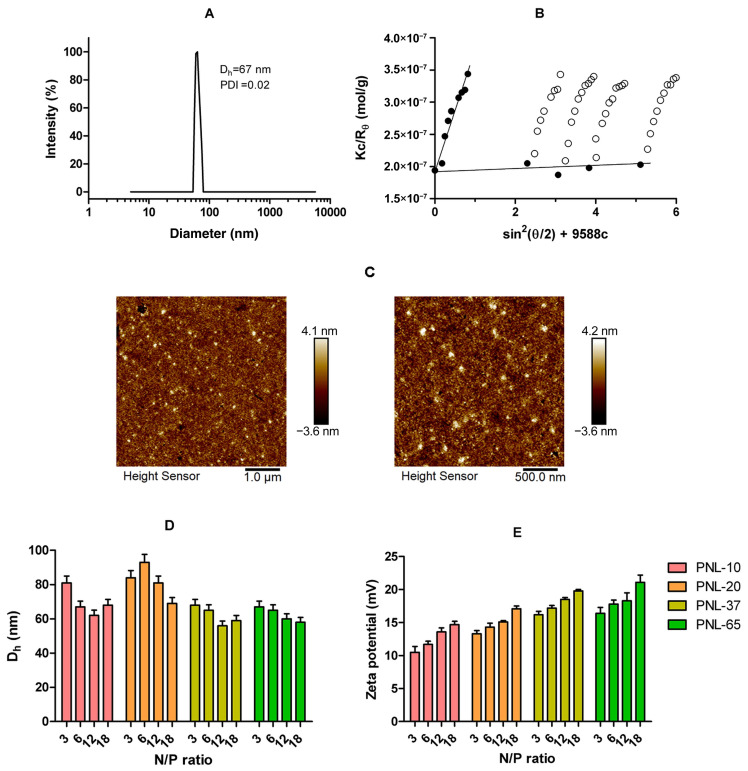
Physicochemical characterization results of the polyplexes. (**A**) Representative particle size distribution measured at an angle of 90° for polyplexes of PNL-65 at N/P 12, prepared by method H-C. (**B**) Zimm plot of polyplexes of PNL-20 at N/P 18, prepared by method H: experimental points (open symbols); extrapolated points to zero concentration and zero scattering angle (closed symbols). (**C**) Representative AFM micrographs of the polyplex particles prepared with PNL-10 at N/P 12, using method H. (**D**) Variations of hydrodynamic diameters and (**E**) ζ potential values of polyplex particles of PNL-10, PNL-20, PNL-37, and PNL-65 with N/P. The polyplexes were prepared by the method RT. Measurements were performed at 25 °C.

The precise structure and composition of the formulations were determined by light scattering measurements and corroborated by atomic force microscopy. Dynamic, electrophoretic, and static light scattering experiments were performed to characterize the complexes of the copolymers with pDNA. In the investigated range of N/P ratios, and for all preparation protocols, monomodal particle size distributions from DLS were typically observed. A representative particle size distribution is shown in Figure 3A.

The PDI values were typically below 0.2, implying formation of nearly uniform particles. Bimodal distributions, containing additional modes of low amplitudes (below 15% accounted at an angle of 90°) were only occasionally (5 out of 48) observed.

The static light scattering data were treated using the Zimm plot method. A typical plot is shown in Figure 3B, whereas the static light scattering parameters—the weight-average molar mass (M_w_), radius of gyration (R_g_), and second virial coefficient (A_2_)—are collected in Table 3 for the complexes of PNL-20, PNL-37, and PNL-65 at different preparation protocols and in Table S2 for the rest of the polyplexes.

The variations of the polyplex particle size with copolymer composition (the length of PLL), preparation protocol, and N/P ratio are presented in Figure 3D and Figure S3. As seen, the size (diameter) of the polyplex particles is in the 60–90 nm range, being somewhat smaller for the formulations of the copolymers PNL-37 and PNL-65 (that is, those with a longer PLL block). Apparently, the copolymers exhibited a divergent complexation behavior with pDNA compared to that with salmon sperm DNA, as previously reported [49]. While larger complexes were formed with salmon sperm DNA, pDNA complexation yielded smaller particles, independent from the preparation protocol. This difference may be attributed to both the differences in topology and length between pDNA and salmon sperm DNA, as observed with quaternized starlike poly(2-(dimethylamino)ethyl methacrylate) [57] and poly(2-(dimethylamino)ethyl methacrylate)-*block*-poly(n-butyl methacrylate) [58], and the distinct preparation environment (HEPES-buffered glucose vs. water).

The results obtained from the electrophoretic light scattering revealed slightly to moderately positively charged particles (Figure 3E and Figure S3). The zeta potential increased with the N/P ratio for all formulations and all methods of preparation, as can be anticipated. The polyplex particles of PNL-37 and PNL-65 are more positively charged (15–20 mV) compared to those of PNL-10 and PNL-20 (10–18 mV).

Evident from the results in Table 3 and Table S2 are the values of M_w_, ranging from 3.5 × 10^6^ to 32.8 × 10^6^ g/mol, typically positive second virial coefficients, indicating favorable particle–solvent interactions, and R_g_ values that generally followed the variations of the hydrodynamic radius, R_h_. The combination of R_g_ and R_h_ gives the dimensionless parameter R_g_/R_h_, which is indicative of particle geometry. The values of R_g_/R_h_ typically ranged between 1.2 and 1.8 with somewhat deviating values below 0.9 and above 2.0, which could be attributed to either slight underestimation, due to the small size of the polyplex particles, or overestimation of R_g_, due to dispersity of the systems.

**Scheme 1:**
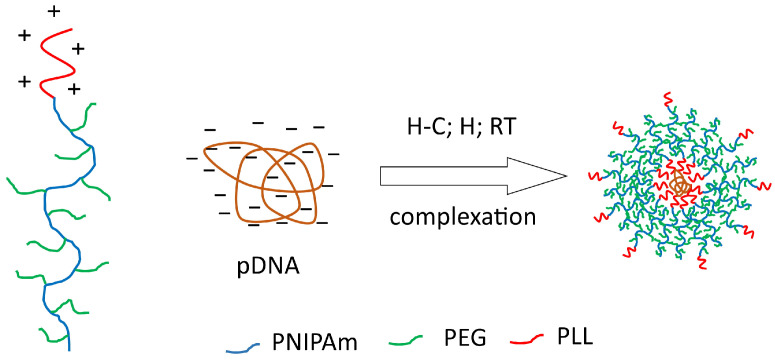
Schematic presentation of the structure of polyplex particles of (PNIPAm)_77_-*graft*-(PEG)_9_-*block*-(PLL)_z_ copolymers with pDNA.

For particles with R_g_/R_h_ in the 1.3–1.7 range, one can predict a morphology which is consistent with that of spherical polymer micelles—that is, particles with a relatively compact and dense core and thick corona. Indeed, the spherical morphology of the polyplex particles was confirmed by AFM observations (Figure 3C). In analogy to this morphology, we can speculate on the polyplex particle structure consisting of a relatively dense interior, resulting from attractive interactions of the oppositely charged PLL segments and pDNA molecules, and a thick corona built mostly of PNIPAm and PEG chains. Noteworthily, the moderately positive ζ potential indicated that some segments of the PLL chains were not involved in interactions with pDNA and possibly remained in the corona. The polyplex particle structure is schematically presented in Scheme 1. The molar mass of the polyplex particles was the most strongly influenced by the N/P: a ca. 3–7-fold increase of M_w_ with decreasing N/P, from 18 to 3, was found for all formulations and methods of preparation, whereas the effects of preparation protocols and copolymer composition were less pronounced, if any.

Another parameter that was extracted from the LS data was the density, ρ, of the material within the polyplex particles. The latter was calculated from the M_w_ and hydrodynamic volume (hence, size) data, assuming a spherical morphology of the particles. The resulting values are listed in Table 3 and Table S2. Given the stronger variations of M_w_ with N/P (the lower the N/P, the higher the molar mass) compared to size variation, ρ was found to range in the 0.040–0.339 g/mL interval and strongly increase with decreasing N/P. In general, the particle density at lower N/P is lower than the density of polyelectrolyte complexes (normally in the 0.3–0.7 g/mL range [59]), which can be easily rationalized in terms of the presence of a bulky, hydrophilic, and inert, with regard to electrostatic interactions with pDNA PNIPAm-*graft*-PEG block building the hydrated corona. At a higher N/P, the polyplex particles exhibited density that was comparable and frequently lower than that of the familiar polymer micelles, implying that the polyplex particles, even the polyelectrolyte core in which the pDNA is condensed, contained appreciable amounts of solvent (water).

Finally, we determined the composition of the polyplexes, assuming that (i) pDNA molecules are an integral and non-dividable part of the polyplexes, and (ii) all components (copolymer and pDNA) are incorporated in the polyplex particles. The results, expressed as the maximal number of pDNA molecules per polyplex particle, are presented in Table 3 and Supporting Table S2. Seemingly, the polyplex particles at higher N/P ratios are composed of only one molecule of pDNA, whereas those at lower N/P ratios are composed of several (up to 10, typically 4–7) pDNA molecules. The estimated number of copolymer molecules per polyplex particle was significantly larger (from tens to 160) and did not depend systematically on the N/P. These findings are fairly consistent with studies reporting an average of 3.5 plasmids per polyplex [60] and 16–30 pDNA copies per considerably larger in size and molar mass polyplex particles [61].

AFM was used to visualize the particles. AFM micrographs (Figure 3C) revealed a mostly spherical morphology of the particles for all formulations. Objects with morphology deviating from the spherical one were occasionally observed. All groups of polyplexes exhibited a size comparable to those determined by light scattering. In conformity with the inherent size dispersity, particles with dimensions somewhat smaller or larger than the average ones were also detected.

### 3.4. Polyanionic Stress Resistance of the Copolymer–pDNA Complexes

**Figure 4:**
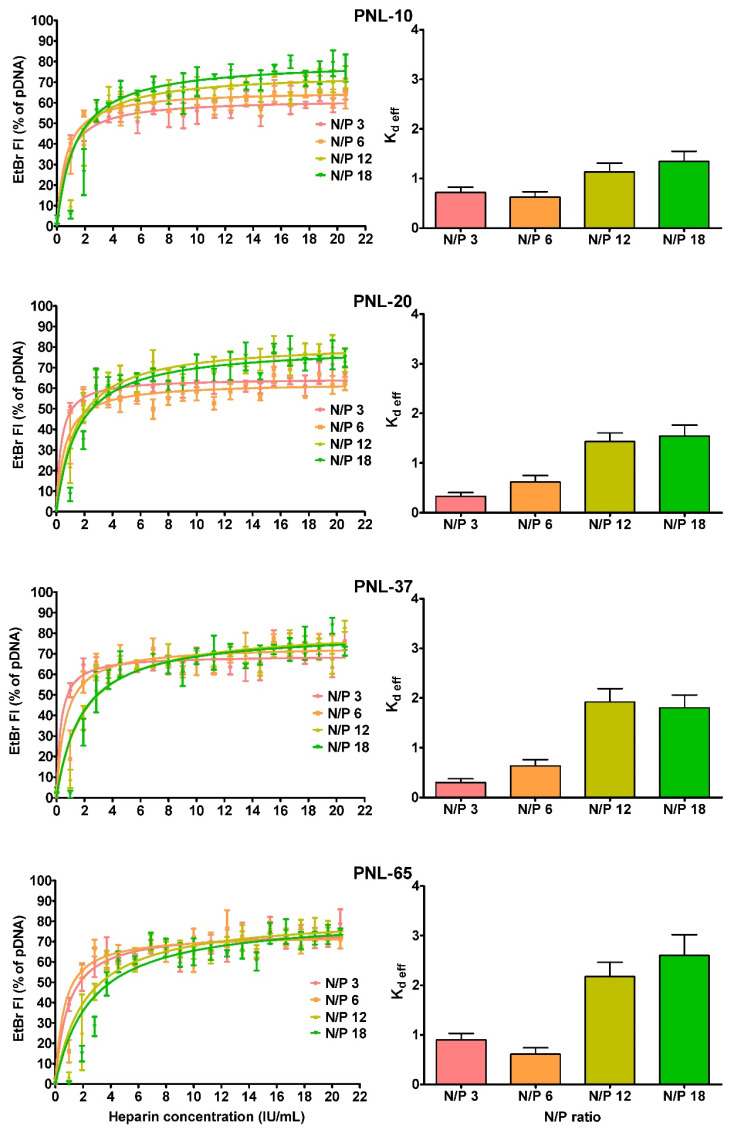
Polyanionic stress resistance of copolymer–pDNA complexes at 3, 6, 12, and 18 N/P ratios. The % change in normalized EtBr fluorescence intensity as the result of increasing heparin concentration is shown in the graphs. For each copolymer, the K_d eff_ values are plotted against the N/P ratio. Polyplexes were prepared at RT for 40 min, as described in the section for polyplex preparation. Following polyplex formation, samples were transferred to a 96-well plate, and an automated heparin titration was performed, with ethidium bromide fluorescence intensity measured after each addition.

Glycosaminoglycans, abundant in biological fluids [62], are negatively charged macromolecules due to sulfate and carboxylate groups on their disaccharide backbones. These anionic macromolecules can destabilize polymer–nucleic acid complexes by competing with nucleic acids for electrostatic binding to cationic polymers [63]. To assess the anionic stress resistance of copolymer–pDNA complexes, an EtBr exclusion assay was performed with increasing heparin concentrations (Figure 4).

The correlation between increasing heparin concentration and elevated EtBr fluorescence (normalized to free pDNA) in polyplex samples demonstrated complex destabilization. Anionic stress resistance increased with the N/P ratio, as evidenced by the change in EtBr fluorescence. Effective dissociation constants (K_d eff_), calculated from parabolic binding isotherm analysis of fluorescence data, demonstrated a direct correlation between K_d eff_ and the N/P ratio. This indicates that higher N/P ratios require increased heparin concentrations for pDNA release. Furthermore, the poly(L-lysine) chain length significantly impacted anionic stress resistance, with K_d eff_ values at N/P 18 increasing from 1.34 (PNL-10) to 2.60 (PNL-65).

### 3.5. Serum Stability of the Copolymer–pDNA Complexes

**Figure 5:**
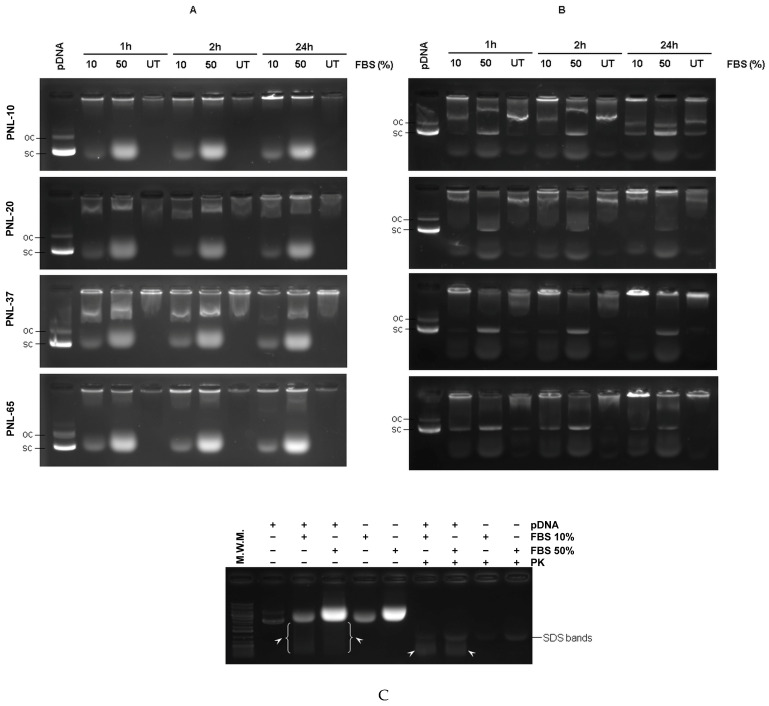
Agarose gel images of the serum stability experiments with the polyplexes prepared at a N/P ratio of 18. The polyplex destabilization study was carried out by incubation of the polyplexes with 10% and 50% FBS for 1 h, 2 h, and 24 h (**A**). pDNA integrity was evaluated after forced decomplexation of the polyplexes by treatment with the cocktail containing proteinase K, heparin, and SDS (**B**). Naked pDNA was treated with FBS/PK to demonstrate the effect of serum nucleases on the unprotected nucleic acid payload (**C**). pDNA served as a positive control of free pDNA in all gels. White arrows show the degraded pDNA smears. Abbreviations: M.W.M—molecular weight marker (Gene Ruler 1 kb plus DNA ladder); ***oc***—open circular conformation of the plasmid; sc—supercoiled conformation of the plasmid; PK—forced release cocktail containing 50 mM disodium EDTA, 2.2 mg/mL proteinase K, 5% SDS, and 900 IU/mL heparin.

The stability of polyplexes at N/P ratios of 6, 12, and 18 was assessed by incubating them with non-inactivated FBS for varying durations (Figure 5). pDNA exists in several topological forms such as supercoiled (*sc*), linear (*L*), and open circular (*oc*). The *sc* pDNA has a smaller hydrodynamic radius compared to the *L* or *oc* forms. Therefore, it is considered the most compact and biologically active form, where the pDNA helix is twisted upon itself and can diffuse faster to the nucleus [64,65,66,67]. The *L* form arises from double-strand breaks in the circular molecule and is more susceptible to nuclease degradation and generally less efficient for gene expression. The *oc* form (also known as nicked DNA) contains one or more single-strand breaks, resulting in a relaxed circular structure which is also less efficient for gene expression compared to the *sc* form. Therefore, these latter two topology forms are less preferred for gene delivery, and the non-viral vectors are expected to maintain the *sc* form of pDNA in physiological fluids [7,64,66]. Direct visualization of pDNA bands after incubation with FBS was obscured by broad, bright bands attributed to serum proteins (Figure 5A,C). Critically, the absence of a low-migrating smear, indicative of degraded pDNA (Figure 5C, white arrows), suggests effective protection of pDNA within the complexes. To further evaluate pDNA integrity, complexes were subjected to forced decomplexation following FBS treatment. Digestion of FBS proteins by proteinase K revealed intact supercoiled pDNA bands (Figure 5B), confirming pDNA protection in its active form. While some exposed pDNA remained trapped in the gel wells [68], particularly in untreated and 10% FBS-treated samples, suggesting potential higher-order interactions with the release cocktail, the observation of intact *sc* pDNA bands after 24 h of FBS exposure, compared to the rapid degradation of naked pDNA, demonstrates robust protection against physiological nucleases. Similar results for both decomplexation and pDNA integrity were observed for lower N/P ratios with all copolymers, as shown in Figures S4 and S5.

### 3.6. In Vitro Transfection Efficiency and Cytotoxicity of the Copolymer–pDNA Complexes

**Figure 6:**
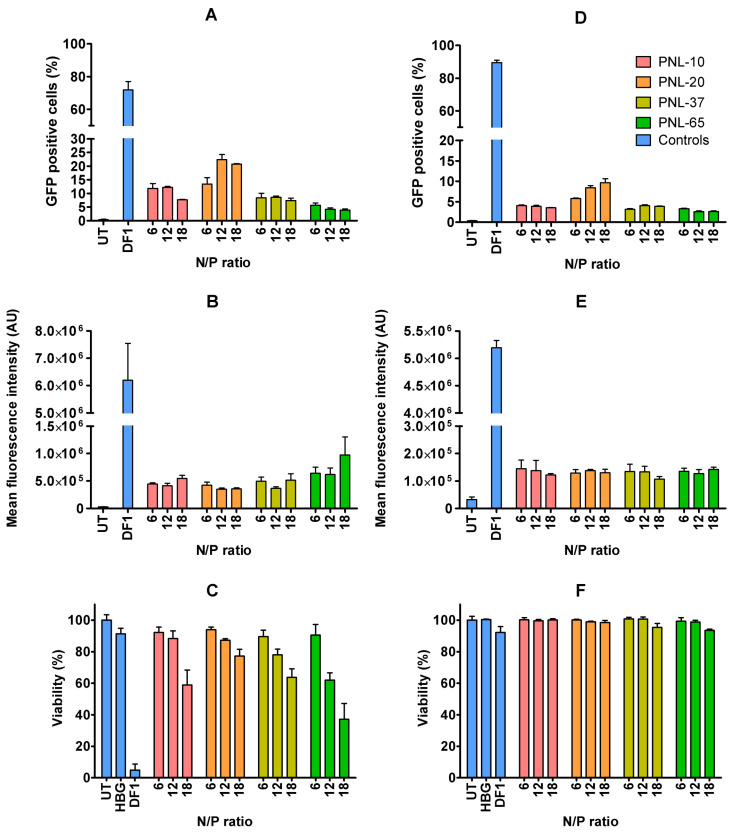
pDNA transfection efficiency of the polymers on PC3 (**A**–**C**) and H1299 (**D**–**F**) cell lines, showing the % of GFP-positive cells, mean fluorescence intensity within the transfected population, and the % of cell viability. UT—untreated cells; HBG—HEPES-buffered glucose buffer-treated cells; DF1—commercial transfection control (DharmaFECT 1). Representative scatter plots from flow cytometry analyses are presented in Figures S9 and S10. The copolymers’ codes and symbols are given in the upper-right-hand graphic.

Initial GFP fluorescence screening identified PC3 and H1299 cells as exhibiting the highest transfection efficiency with the copolymers, while HCC827, HaCaT, and SK-MEL-30 cells showed minimal transfection. We performed preliminary tests to determine the effect of the preparation method on the transfection efficiency of the polyplexes on PC3 cells (Figure S6). There were no differences in the transfection efficiency across distinct preparation methods. Therefore, subsequent experiments were conducted using polyplexes prepared by method RT. Subsequent experiments focused on PC3 and H1299, quantifying transfection efficiency via flow cytometry (Figure 6).

PNL-20 demonstrated the highest N/P ratio-dependent transfection efficiency in both cell lines (Figure 6A,D). While PNL-65 exhibited the lowest transfection efficiency, it yielded the highest fluorescence intensity within the transfected PC3 population and equivalent intensity in H1299 (Figure 6B,E). The case of low transfection efficiency accompanied by a high fluorescence intensity has been previously observed for both pDNA and mRNA with various transfection methods [69,70], and implies that the transgene expression level within the transfected population is high. Fluorescence microscopy observations corroborated well with the flow cytometry results and pretreatment of the polyplexes did not lead to a great reduction in transfected cells, as was observed for the positive commercial transfection control (Figures S7 and S8). While polyplexes formed by copolymers with a higher charge density (PNL-37 and PNL-65) demonstrate high anionic stress resistance, their transfection efficiency is not as high as that of copolymers with a lower charge density (PNL-10 and PNL-20). These data show that loose polyplexes (those with a lower effective dissociation coefficient and lower density) are better able to release pDNA upon interaction with cytosolic polyanions. Maury et al. showed that lower EtBr fluorescence quenching in histidinylated linear PEI polyplexes indicates looser formation, enabling pDNA access for transcription in non-proliferative cells [56], whereas Lau et al. related the less compact polyplex structure with improved structural dynamics and better gene silencing efficiency [71]. This implies that transfection efficiency is governed by a balance between the complex strength and its ability to release the pDNA under anionic stress [72]. Light scattering (LS) data, on the other hand, revealed that PNL-10 and PNL-20 formed less tightly structured polyplexes than PNL-37 and PNL-65. Interestingly, despite LS data indicating a similarly loose overall structure to PNL-20, dye exclusion results showed that PNL-10 actually displayed the highest EtBr fluorescence quenching at N/P ratios below 1.3 and, notably, all copolymers displayed over 90% quenching above this N/P ratio. This apparent discrepancy might arise because LS and EtBr quenching are sensitive to different aspects of polyplex architecture. While LS provides information about the overall size and density of the particles [73], EtBr quenching specifically reflects the accessibility of the DNA to the dye within the complex [50,56]. Therefore, PNL-10 polyplexes could possess a generally loose overall structure (as suggested by SLS) but still exhibit regions of DNA that are less accessible to EtBr, leading to higher quenching compared to PNL-20. These findings collectively suggest that the correlation between looser polyplex formation (as indicated by SLS) and high transfection efficiency (observed for PNL-20) might be more complex than a simple inverse relationship with EtBr quenching alone, particularly given PNL-10’s high quenching despite its loose overall structure. Cytotoxicity assays in PC3 cells revealed increasing toxicity with the N/P ratio, with PNL-20 exhibiting the lowest toxicity (viability > 80%). H1299 cells showed minimal cytotoxicity (viability > 90%) across all N/P ratios (Figure 6C,F). Usually, the observed correlation between high transfection efficiency and cytotoxicity, a common trade-off in transfection reagents [74,75,76], is being addressed through polymer structure modifications. While PNL-20 did not exhibit transfection efficiency superior to the commercial reagent DharmaFECT 1, its significantly lower cytotoxicity profile on PC3 cells at N/P 18 (Figure 6C) positions it as a more promising and suitable candidate for further development, particularly for in vivo applications where biocompatibility is of utmost importance.

To investigate the efficiency of the copolymers in facilitating endo-lysosomal degradation escape, a lysosomal membrane destabilization assay was performed. AO, a basic dye with a weak lysosomotropic property, passively accumulates in lysosomes [54,77]. Once inside, AO molecules become protonated and assemble into dimers, preventing their diffusion back out of the acidic vesicles. In this acidic environment, AO exhibits red-to-orange fluorescence, whereas in the cytoplasm and nucleus, it fluoresces green [54,78]. The intensity of AO red fluorescence directly indicates lysosomal integrity.

**Figure 7:**
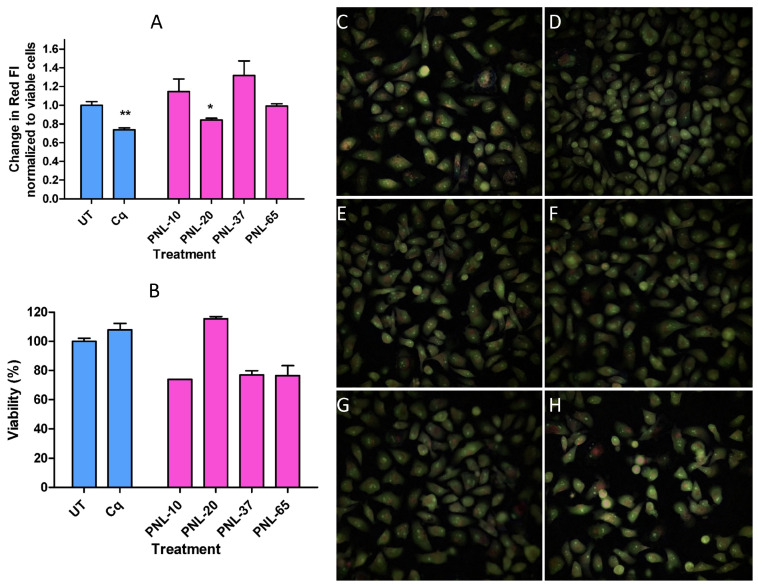
Lysosomal membrane destabilization by polyplexes prepared with the copolymers. Cells were treated with the polyplexes for 24 h and stained with AO for 15 min at 37 °C. The intensity of the red fluorescence that indicates the AO in acidic lysosomes is shown in (**A**). The viability of cells during the treatments for the lysosomal membrane destabilization assay is shown in (**B**). Representative fluorescence microscopy images of cells are displayed in (**C**–**H**) (untreated cells, Cq-treated cells, PNL-10-, PNL-20-, PNL-37-, and PNL-65-treated cells, respectively); fluorescence and viability results are presented as mean ± SD. UT—untreated cells, Cq—chloroquine-treated cells. * *p* < 0.05; ** *p* < 0.01.

Among the copolymers tested, only PNL-20 provided significant lysosomal membrane destabilization, as observed through fluorescence measurements (Figure 7A).

As previously reported by Fornaciari et al., AO can lead to cell toxicity after prolonged exposure and under light illumination [77]. Therefore, we also evaluated cytotoxicity under the experimental conditions to normalize the AO red signal for live cells (Figure 7B). Polyplexes prepared with PNL-20 did not show toxicity under these conditions, while the remaining polyplexes did. Representative fluorescence microscopy results further confirm the decrease in red fluorescence (Figure 7C–H). These findings indicate that PNL-20 destabilizes lysosomal membranes more effectively than the other three copolymers, thereby facilitating endosomal escape. Collectively, these results demonstrate that PNL-20 is the best performer among the tested copolymers.

## 4. Conclusions

In summary, the cytotoxicity of (PNIPAm)_77_-*graft*-(PEG)_9_-*block*-(PLL)_z_ copolymers was primarily determined by the PLL block length, exhibiting minimal toxicity at transfection concentrations. The method of polyplex preparation did not affect pDNA condensation. Physicochemical analyses confirmed strong N/P ratio-dependent pDNA binding and the formation of spherical, core–corona structured polyplexes. Higher N/P ratios resulted in smaller polyplexes (1–2 pDNA molecules per polyplex), while lower ratios yielded slightly larger ones (up to 10 pDNA molecules) with densities comparable to polymer micelles. Polyplex compactness, assessed by the density of the material within the particles, was inversely related to PLL block length (PNL-10 and PNL-20 being less tight than PNL-37 and PNL-60). Resistance to anionic stress was directly correlated with the PLL block length and N/P ratio, while protection of pDNA from nuclease degradation was seen at all N/P ratios. Notably, PNL-20 demonstrated the highest transfection efficiency, suggesting an optimal balance between polyplex tightness and plasmid release. PNL-20 also demonstrated significant lysosomal membrane destabilization. Further investigation into the specific mechanisms of cellular internalization and intracellular trafficking of polyplexes prepared with (PNIPAm)_77_-*graft*-(PEG)_9_-*block*-(PLL)_z_ copolymers is necessary. However, the low in vitro toxicity of these copolymers warrants further exploration of their potential for in vivo nucleic acid delivery in disease models.

## Figures and Tables

**Table 1:** Characterization data of the (PNIPAm-*graft*-PEG)-*block*-PLL copolymers.

Sample Code	Copolymer Composition	Mn ^a^ (g/mol)	Mn ^b^ (g/mol)	Đ ^b^	DP of PLL Block ^a^
PNL-10	(PNIPAm)_77_-*graft*-(PEG)_9_-*block*-(PLL)_10_	15,200	6300	1.23	10
PNL-20	(PNIPAm)_77_-*graft*-(PEG)_9_-*block*-(PLL)_20_	17,300	6800	1.23	20
PNL-37	(PNIPAm)_77_-*graft*-(PEG)_9_-*block*-(PLL)_37_	20,800	5200	1.18	37
PNL-65	(PNIPAm)_77_-*graft*-(PEG)_9_-*block*-(PLL)_65_	26,700	5800	1.19	65

^a 1^H NMR in D_2_O. ^b^ Gel permeation chromatography in 0.4 mol % formic acid aqueous solution, using poly(2-vinylpyridine) standards.

**Table 2:** IC_50_ values of the copolymers on the tested cell lines.

Copolymer	IC_50_ on the Corresponding Cell Line (µg/mL)									
	PC3		H1299		HCC827		SK-MEL-30		HaCaT	
	48 h	72 h	48 h	72 h	48 h	72 h	48 h	72 h	48 h	72 h
**PNL-10**	173.4	195.6	132.8	142.6	193.0	199.0	89.02	94.03	161.2	149.1
**PNL-20**	97.87	105.5	90.08	87.29	77.20	85.03	55.19	50.00	120.1	120.4
**PNL-37**	68.22	81.49	57.09	51.05	66.41	61.95	30.25	25.40	57.51	51.03
**PNL-65**	47.30	54.18	37.46	41.68	50.99	57.25	28.72	27.85	45.51	45.75

**Table 3:** Static light scattering characterization data, number of pDNA copies, and density of material in polyplex particles prepared with PNL-20, PNL-37, and PNL-65 at N/P = 3–18, and different complexation methods.

Copolymer/ Method	N/P	R_g_ (nm)	R_g_/R_h_	10^4^ × A_2_ (mL·mol/g^2^)	10^−6^ × M_w_ (g/mol)	pDNA per Complex	ρ (g/mL)
**PNL-20/H**	3	48.7	1.30	−0.75	16.1	4	0.121
	6	46.0	1.30	0.48	10.7	3	0.096
	12	61.4	1.73	1.54	5.3	1	0.033
	18	62.7	1.79	0.15	5.2	1	0.048
**PNL-37/RT**	3	42.9	1.26	0.40	23.5	7	0.237
	6	45.0	1.38	4.97	11.5	3	0.132
	12	48.2	1.72	−0.10	6.6	2	0.119
	18	39.0	1.32	2.60	4.1	1	0.063
**PNL-65/H-C**	3	41.4	1.24	0.70	23.7	7	0.253
	6	42.7	1.24	3.70	12.1	3	0.117
	12	46.0	1.39	3.80	6.2	1	0.068
	18	45.0	1.34	3.80	4.6	1	0.048

## Data Availability

Data are contained within this article.

## Supplementary Materials

The following supporting information can be downloaded at https://www.mdpi.com/article/10.3390/pharmaceutics17081012/s1: Table S1. Characterization data of the PNIPAm-g-PEG macroinitiator. Scheme S1. Synthetic route for preparation of (PNIPAm-*graft*-PEG)-*block*-PLL copolymers. Table S2: Static light scattering characterization data, number of pDNA copies, and density of material in particles prepared with PNL-10, PNL-20, PNL-37, and PNL-65 at N/P = 3–18, using different complexation methods. Figure S1: Ethidium bromide fluorescence in the presence of the copolymers. PNL-10 and PNL-20 were tested. Copolymer samples were prepared by serial dilutions in HBG buffer. Two series were prepared: in the first set, the copolymers were kept at room temperature, and in the second series the copolymer was heated at 65 °C for 5 min. After cooling the heated samples to RT for 5 min, 20 µL from each sample was transferred to triplicate wells of a 96-well plate. A total of 80 µL of 400 ng/mL ethidium bromide was added to each well and incubated for 10 min at RT. Ethidium bromide alone in HBG was tested as the control. Figure S2: pDNA compaction tested on the polyplexes prepared using the method H (A) and the method H-C (B). Figure S3: Particle size and ζ potential variation of polyplexes prepared at four different N/P ratios using three different preparation methods. Measurements were performed at a 90° scattering angle and 25 °C. RT—polyplexes were prepared by mixing and incubating the copolymer and the pDNA at room temperature for 40 min. H—polyplex preparation started by first heating the copolymer solution to 65 °C for 5 min in polypropylene micro centrifuge tubes. While heating, the pDNA solution was added in equal volume and mixed by pipetting. Immediately after mixing, the tube was brought to RT and incubated for 40 min. H-C—polyplex preparation started by first heating the copolymer solution to 65 °C for 5 min and afterwards, bringing it to RT for 5 min. The pDNA solution was then added in equal volume, mixed, and incubated at RT for 40 min. Figure S4: Agarose gel images of the decomplexation study carried out by incubation of the complexes at 6, 12, and 18 N/P ratios with 10% and 50% FBS for 1 h, 2 h, and 24 h. pDNA served as a positive control of free pDNA. Figure S5: Agarose gel images demonstrating the pDNA integrity in the presence of FBS. After the complex formation, the complexes formed at N/P ratios of 6, 12, and 18 were incubated with 10% and 50% FBS for 1 h, 2 h, and 24 h. Subsequently, a proteinase K-, heparin-, and SDS-containing cocktail was added to the samples and incubated at 56 °C for 40 min to destabilize the complexes and liberate the pDNA for electrophoretic analysis. pDNA alone served as a positive control of free pDNA. Figure S6: Preliminary results demonstrating the transfection efficiency of the polyplexes obtained by the three different preparation methods on PC3 cells. Room temperature (RT), Hot complexation at 65 °C (Hot), and complexation with Heated and cooled polymers (Heat–cool). Lipofectamine-2000 and DharmaFECT 1 were used as commercial transfection controls. Figure S7: Fluorescence microscopy images of PC3 cells subjected to 72 h transfection with the polyplexes. Figure S8: Fluorescence microscopy images of H1299 cells subjected to 72 h transfection with the polyplexes. Figure S9: Flow cytometry scatter plots of PC3 cells transfected with polyplexes prepared at three N/P ratios. Cells were treated with the polyplexes for 72 h, as described under Materials and Methods, and analyzed for GFP signal. UT—untreated cells; DF1—DharmaFECT 1 commercial transfection reagent. Figure S10: Flow cytometry scatter plots of H1299 cells transfected with polyplexes prepared at three N/P ratios. Cells were treated with the polyplexes for 72 h, as described under Materials and Methods, and analyzed for GFP signal. UT—untreated cells; DF1—DharmaFECT 1 commercial transfection reagent.
