# Supplementary material for: Plasmid DNA Delivery to Cancer Cells with Poly(L-lysine)-Based Copolymers Bearing Thermally Sensitive Segments: Balancing Polyplex Tightness, Transfection Efficiency, and Biocompatibility
Source: Pharmaceutics. 2025 Aug 2;17(8):1012. doi: 10.3390/pharmaceutics17081012 (PMC12389523; doi:10.3390/pharmaceutics17081012)
Supplement: Supplementary file 1 [file pharmaceutics-17-01012-s001.zip › pharmaceutics-3774027-supplementary-new.pdf]

Supplementary Materials: Plasmid DNA Delivery to Cancer Cells with Poly(L-lysine)-based Copolymers bearing Thermally Sensitive Segments: Balancing Polyplex Tightness, Transfection Efficiency, and Biocompatibility

Mustafa Kotmakci [1,*], Natalia Toncheva-Moncheva [2], Sahar Tarkavannezhad [1], Bilge Debelec Butuner [1], Ivaylo Dimitrov [2] and Stanislav Rangelov [2,*]

[1] *Department of Pharmaceutical Biotechnology, Faculty of Pharmacy, Ege University, Ankara Street 172/98 Campus-Bornova, 35040-Izmir, Türkiye*

[2] *Institute of Polymers, Bulgarian Academy of Sciences, Akad. G. Bonchev St. 103-A, 1113 Sofia, Bulgaria*

Corresponding Authors

*Dr. Mustafa Kotmakci
Department of Pharmaceutical Biotechnology,
Faculty of Pharmacy, Ege University,
Ankara Street 172/98
Campus-Bornova, 35040-Izmir, Türkiye
email: mustafa.kotmakci@ege.edu.tr

*Prof. DSc. Stanislav Rangelov
Institute of Polymers,
Bulgarian Academy of Sciences,
Akad. G. Bonchev St. 103-A,
1113 Sofia, Bulgaria
email: rangelov@polymer.bas.bg

Table S1. Characterization data of the PNIPAm-*graft*-PEG macroinitiator. Adapted from reference [49] with permission from Elsevier.

Macroinitiator composition	M_n [a] (g/mol)	Đ[a]	M_n [b] (g/mol)	$T_{d5\%}$ [c] (°C)	T_{dmax}[d] (°C)	T_{CP} [e] (°C)
$(PNIPAM)_{77}$-*graft*-$(PEG)_9$	8,900	1.48	13,100	115	425	41

[a] GPC in DMF, polystyrene standards. [b] The overall molar mass was estimated through an acid–base titration of the terminal primary amine groups. [c] Initial degradation temperature corresponding to weight loss of 5%. [d] Maximum degradation temperature corresponding to weight loss of 50%. [e] The cloud point temperature (T_{CP}) was determined as 50% reduction of the initial optical transmittance of an aqueous solution of $(PNIPAM)_{77}$-*graft*-$(PEG)_9$ with concentration of 10 mg/ml.

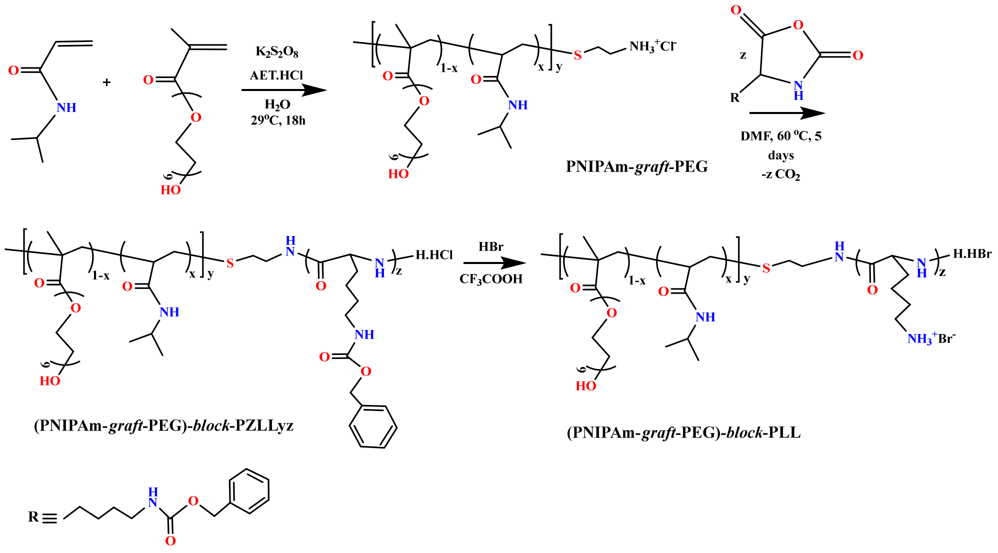

Scheme S1. Synthetic route for preparation of (PNIPAm-*graft*-PEG)-*block*-PLL copolymers. Adapted from reference [49] with permission from Elsevier.

Table S2. Static light scattering characterization data, number of pDNA copies, and density of material in particles prepared with PNL-10, PNL-20, PNL-37, and PNL-65 at N/P = 3 – 18, using different complexation methods.

Copolymer type / Complexation method	N/P	R_g (nm)	R_g/R_h	10^4 x A_2 (mL.mol/g^2)	10^{-6} x M_w (g/mol)	pDNA molecules per complex	ϱ (g/mL)
**PNL-10 / RT**	3	126.0 [a]	3.11[a]	2.55	32.0	10	0.191
	6	73.0 [a]	2.17[a]	1.10	11.6	3	0.121
	12	76.0[a]	2.45[a]	4.50	6.1	1	0.081
	18	58.7	1.73	-0.05	4.0	1	0.040
**PNL-10 / H**	3	95.0[a]	2.71[a]	1.30	16.4	5	0.152
	6	45.8	1.24	1.10	8.2	2	0.065
	12	49.5	1.27	-0.69	7.9	2	0.053
	18	61.8	1.82	1.50	4.1	1	0.041
**PNL-10 / H-C**	3	33.8[b]	0.90[b]	-9.20	15.8	4	0.117
	6	28.8[b]	0.83[b]	-4.00	11.8	3	0.111
	12	59.8	1.84	0.74	7.2	2	0.083
	18	33.8	1.07	-0.40	3.7	1	0.047
**PNL-20 / RT**	3	42.0	1.00	-0.47	17.5	5	0.093[c]
	6	51.9	1.55	0.48	17.2	5	0.181
	12	66.5[a]	2.14[a]	0.62	5.7	1	0.075
	18	55.4	1.63	-1.10	4.9	1	0.049
**PNL-20 / H-C**	3	41.6	0.90	-1.62	20.5	6	0.082[c]
	6	60.0	1.76	1.60	13.0	3	0.130
	12	-	-	-	-	-	-
	18	57.1	1.70	0.45	4.7	1	0.049
**PNL-37 / H**	3	35.0	1.04	-0.04	32.8	10	0.339
	6	62.0	1.88	0.30	17.1	5	0.189
	12	65.0[a]	2.00[a]	2.00	6.1	1	0.071
	18	50.7	1.58	-0.36	4.4	1	0.053
**PNL-37 / H-C**	3	-	-	-	-	-	-
	6	44.0	1.31	1.60	11.3	3	0.118
	12	78.9[a]	2.50[a]	1.10	8.5	2	0.107
	18	38.3	1.20	1.30	4.6	1	0.056
**PNL-65 / RT**	3	42.7	1.27	-0.04	20.4	6	0.213
	6	46.1	1.42	0.70	10.1	3	0.117
	12	64.0[a]	2.13[a]	2.09	5.7	1	0.084
	18	87.0[a]	3.00[a]	8.60	5.6	1	0.091
**PNL-65 / H**	3	40.5	1.31	3.90	14.1	4	0.189
	6	68.7[a]	2.08[a]	-1.10	17.1	5	0.189
	12	37.0	1.30	1.42	4.4	1	0.076
	18	55.4	1.82	0.40	5.8	1	0.0811

The standard deviations are in the 0.5–5.2% range. [a] – overestimation of R_g; [b] – underestimation of R_g; [c] – overestimation of R_h. RT – complexation at room temperature; H – hot complexation method; H-C – heat-cool complexation method.

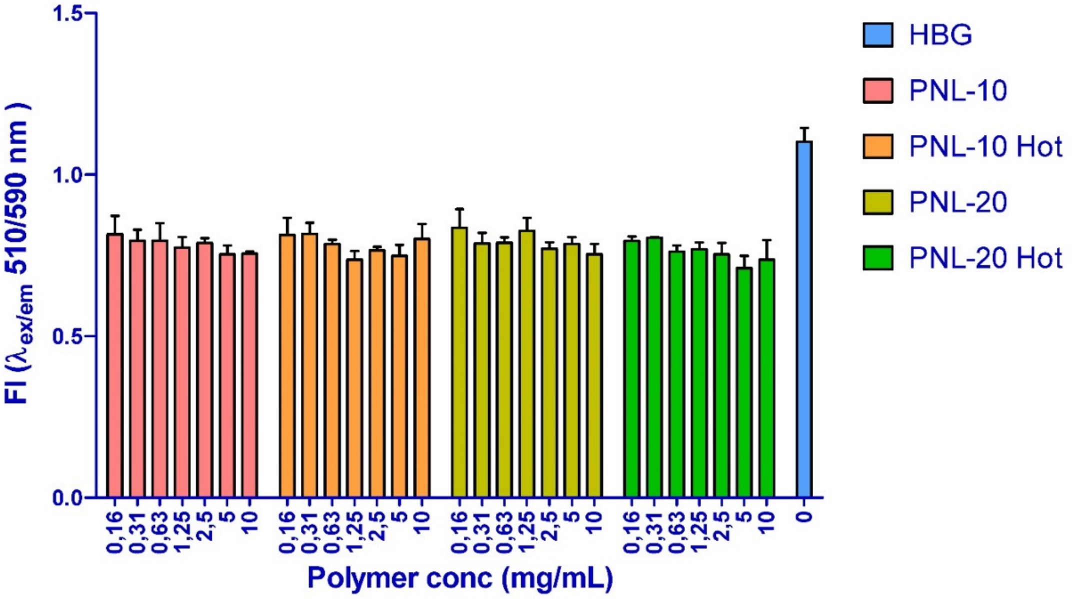

Figure S1. Ethidium bromide fluorescence in the presence of the copolymers. PNL-10 and PNL-20 were tested. Copolymer samples were prepared by serial dilutions in HBG buffer. Two series were prepared, in the first set the copolymers were kept at room temperature, and in the second series the copolymer was heated at 65°C for 5 min. After cooling the heated samples to RT for 5 min, 20 μL from each sample were transferred to triplicate wells of a 96-well plate. 80 μL of 400 ng/mL ethidium bromide were added to each well and incubated for 10 min at RT. Ethidium bromide alone in HBG was tested as the control.

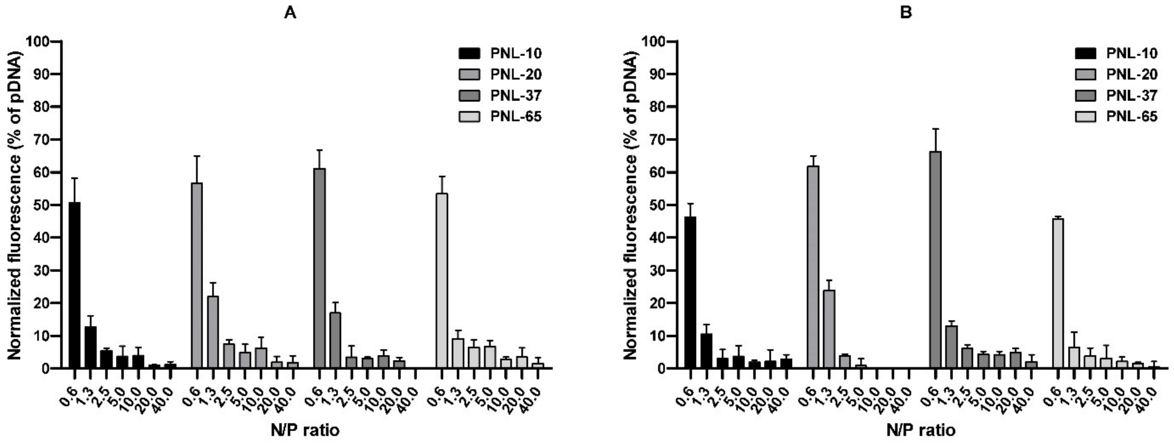

Figure S2. pDNA compaction tested on the polyplexes prepared using the method H (**A**) and the method H-C (**B**).

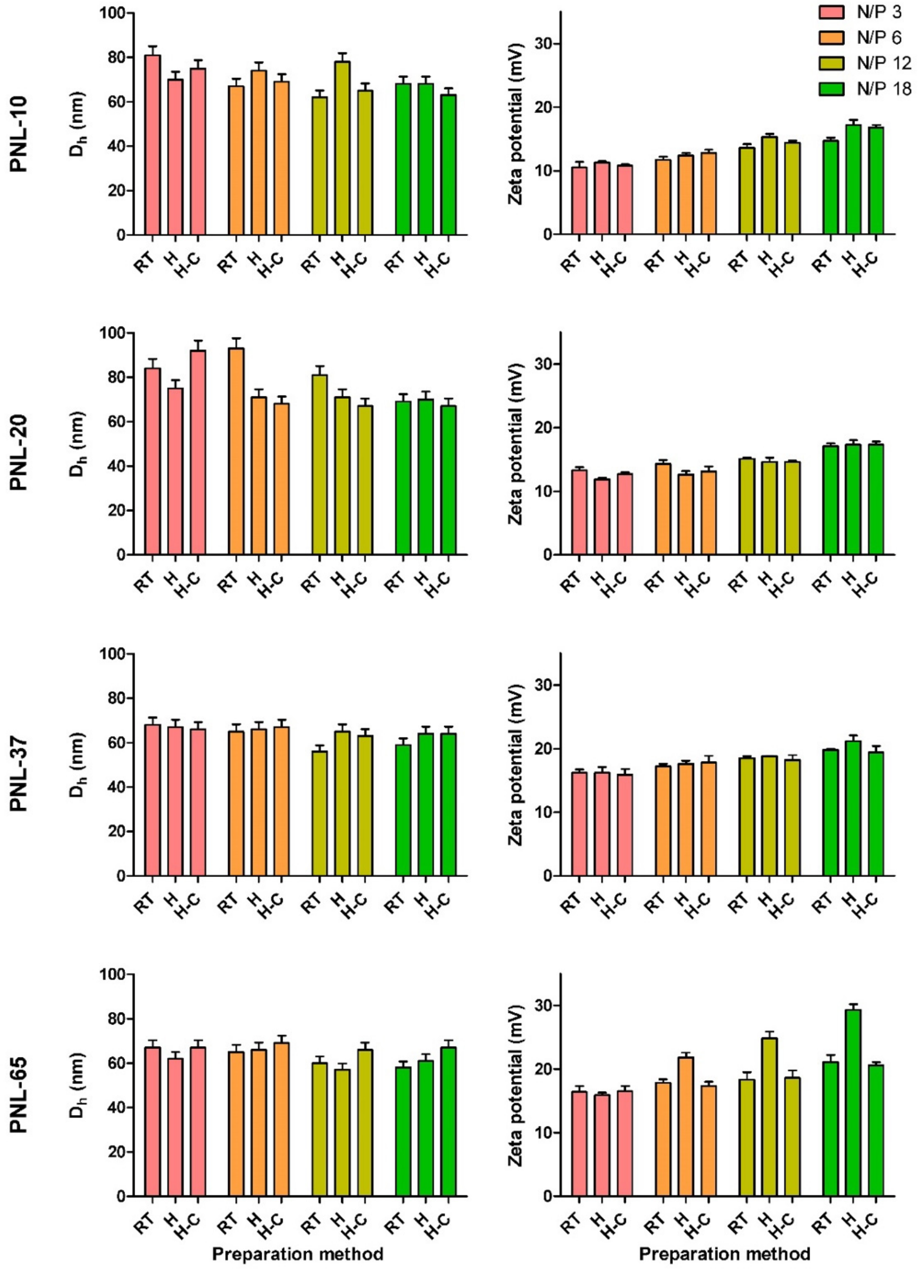

Figure S3. Particle size and ζ potential variation of polyplexes prepared at four different N/P ratios using three different preparation methods. Measurements were performed at 90° scattering angle and 25°C. **RT** – polyplexes were prepared by mixing and incubating the copolymer and the pDNA at room temperature for 40 min; **H** – polyplex preparation started by first heating the copolymer solution to 65°C for 5 min in polypropylene micro centrifuge tubes. While heating, the pDNA solution was added in equal volume and mixed by pipetting. Immediately after mixing the tube was brought to RT and incubated for 40 min; **H-C** – polyplex preparation started by first heating the copolymer solution to 65°C for 5 min and afterwards, bringing it to RT for 5 min. The pDNA solution was then added in equal volume, mixed and incubated at RT for 40 min.

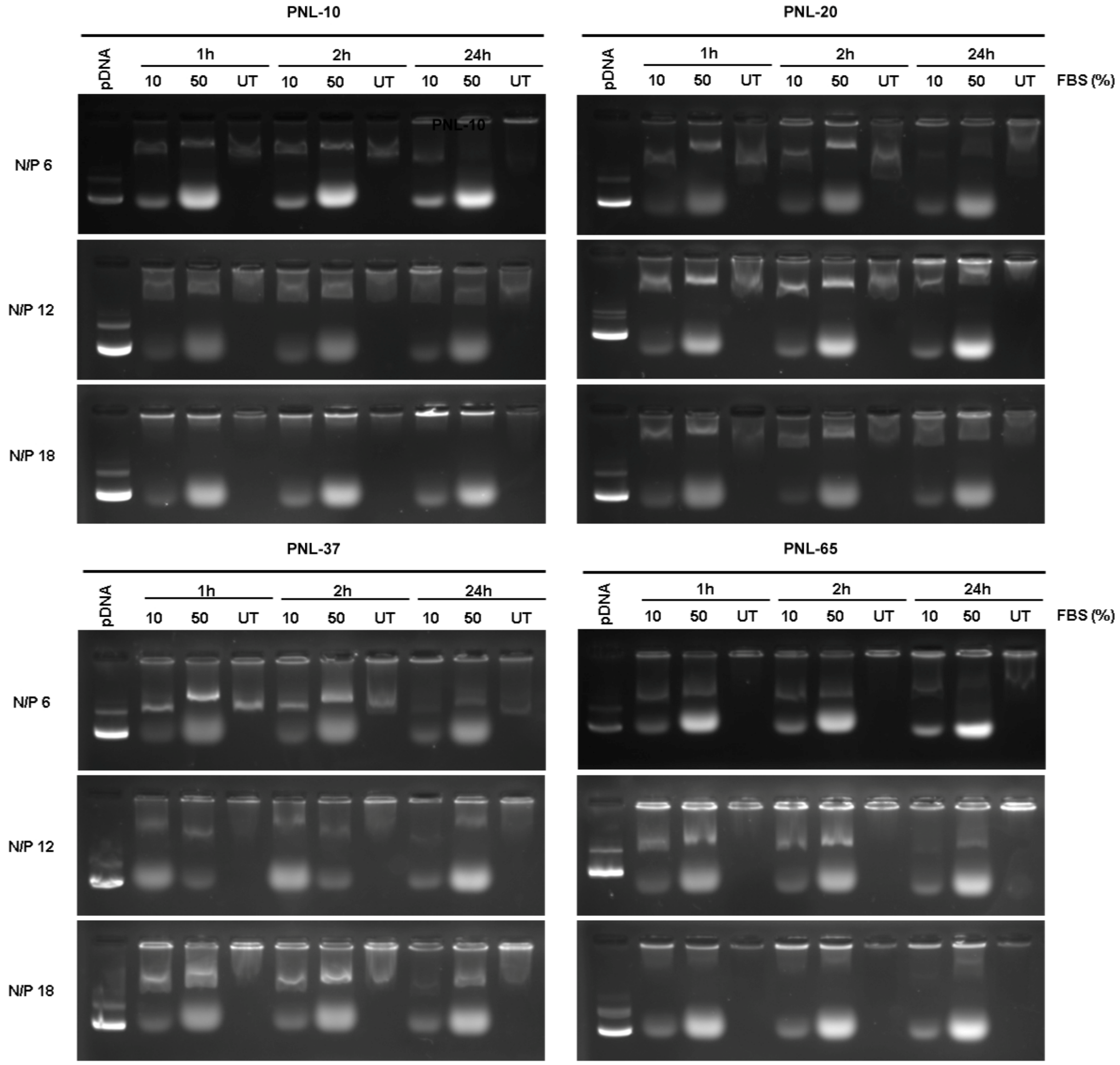

Figure S4. Agarose gel images of the decomplexation study carried out by incubation of the complexes at 6, 12, and 18 N/P ratios with 10% and 50% FBS for 1h, 2h, and 24 hours. pDNA served as a positive control of free pDNA.

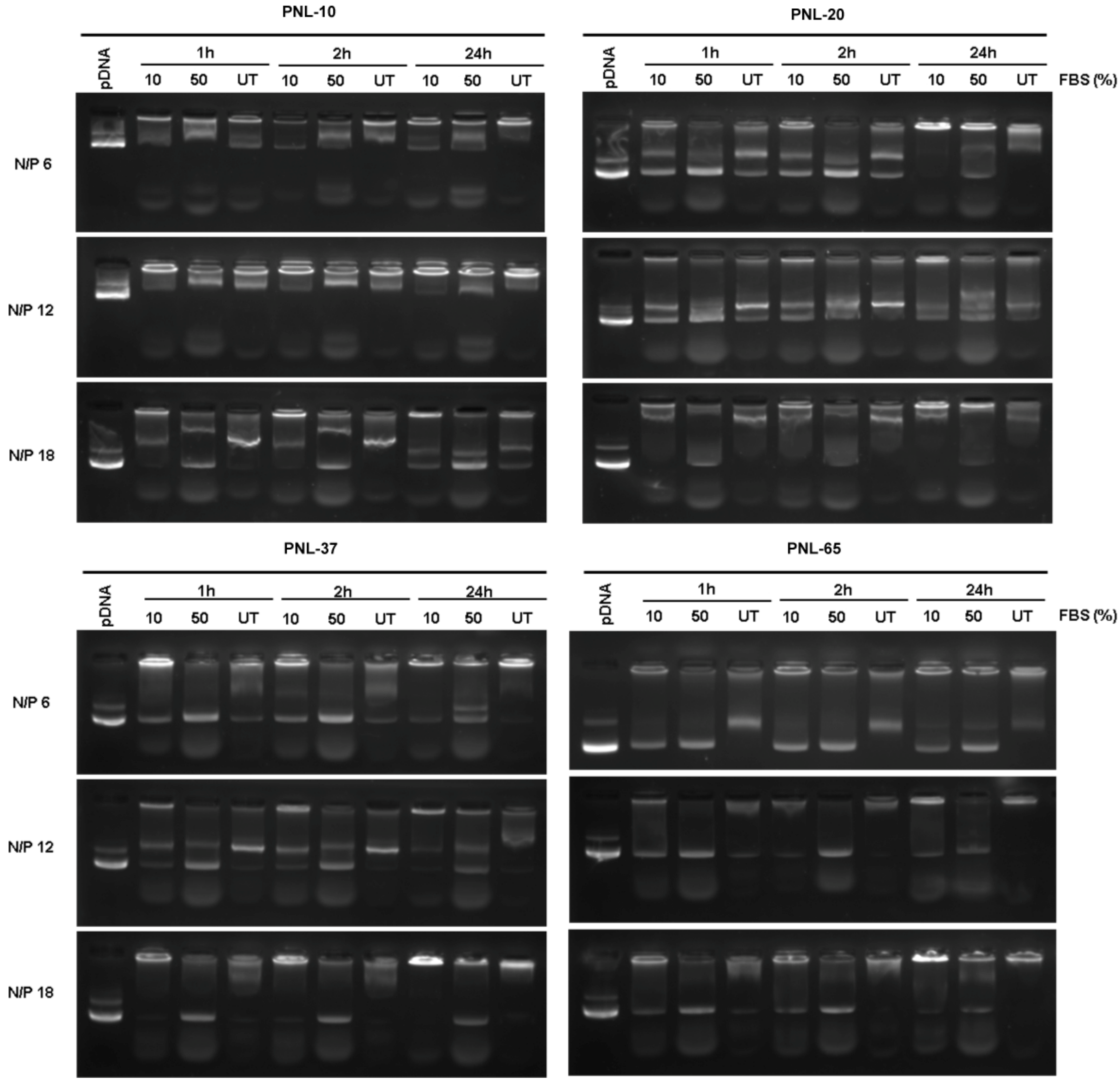

Figure S5. Agarose gel images demonstrating the pDNA integrity in the presence of FBS. After the complex formation, the complexes formed at N/P ratios of 6, 12, and 18 were incubated with 10% and 50% FBS for 1h, 2h, and 24 hours. Subsequently, a cocktail containing proteinase K, heparin and SDS was added to the samples and incubated at 56°C for 40 min to destabilize the complexes and liberate the pDNA for electrophoretic analysis. pDNA alone served as a positive control of free pDNA.

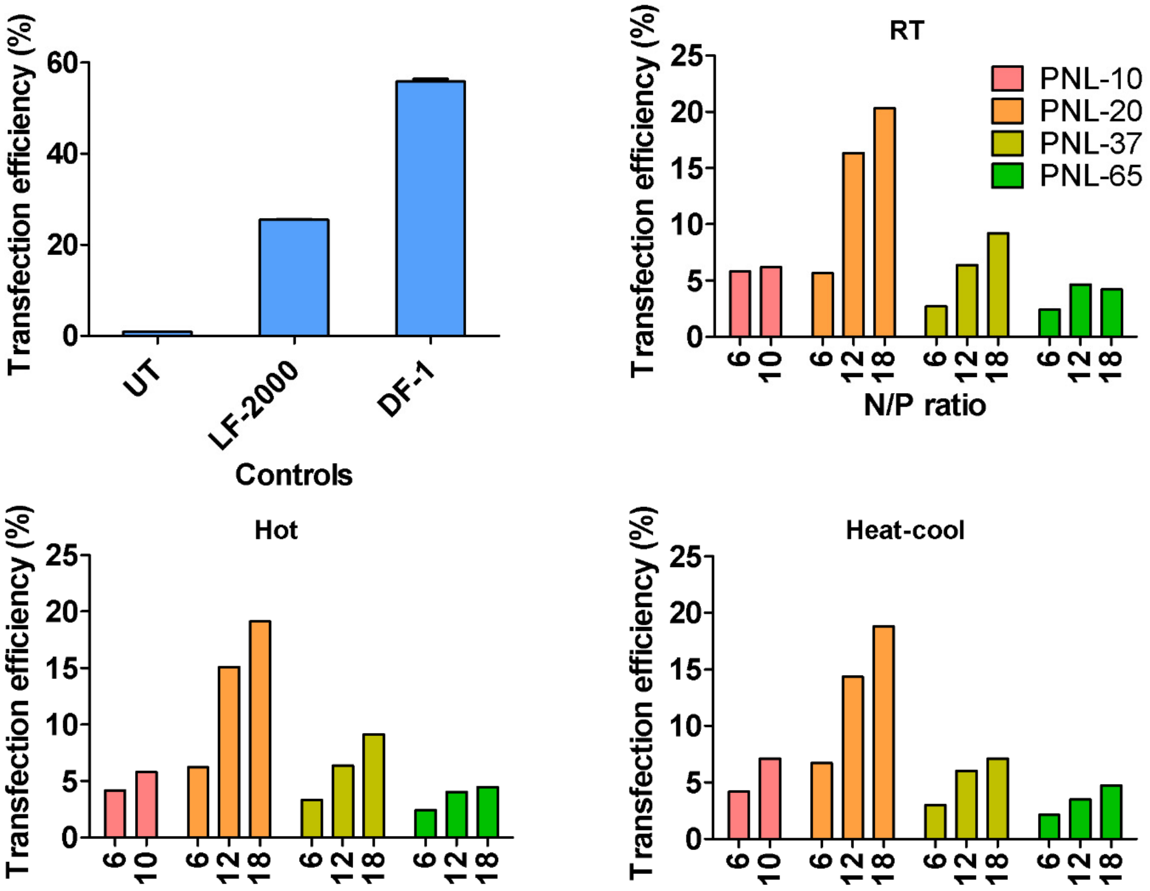

Figure S6. Preliminary results demonstrating the transfection efficiency of the polyplexes obtained by the three different preparation methods on PC3 cells. Room temperature (RT), Hot complexation at 65°C (Hot) and complexation with Heated and cooled polymers (Heat-cool). Lipofectamine-2000 and DharmaFECT 1 were used as positive commercial transfection controls.

FBS treatment of the polyplexes and cell transfection

Complexes were prepared as described in the main text. After the complexation, active FBS was added to the complexes in 1:1 v/v ratio, mixed 10 times by pipetting up and down and incubated at 37°C. Cells were seeded in 24-well plates at a density of 6 x 10^4 cells/well in 500 µL of complete growth medium. The following day, at least 30 minutes prior to treatment, the culture media of normal complex application group were replaced with 450 µL of fresh complete growth medium. To ensure that the transfection in all groups takes place under equal conditions, the medium of cells to be treated with FBS-pretreated polyplexes were replaced with 450 µL of fresh growth medium supplemented with 5% FBS. In this way, both treatment groups were incubated in media containing equal amount of FBS. Cells were treated with the complexes at a pDNA concentration of 1 µg/mL. The treatment volume constituted 10% of the total culture medium volume for all experimental groups. HBG treatment served as a vehicle control. DharmaFECT 1 (Horizon Discovery) was used as a positive control according to the manufacturer's protocol. Transfection was performed for 72 hours. Following incubation, GFP expression in transfected cells was visualized under the fluorescence microscope.

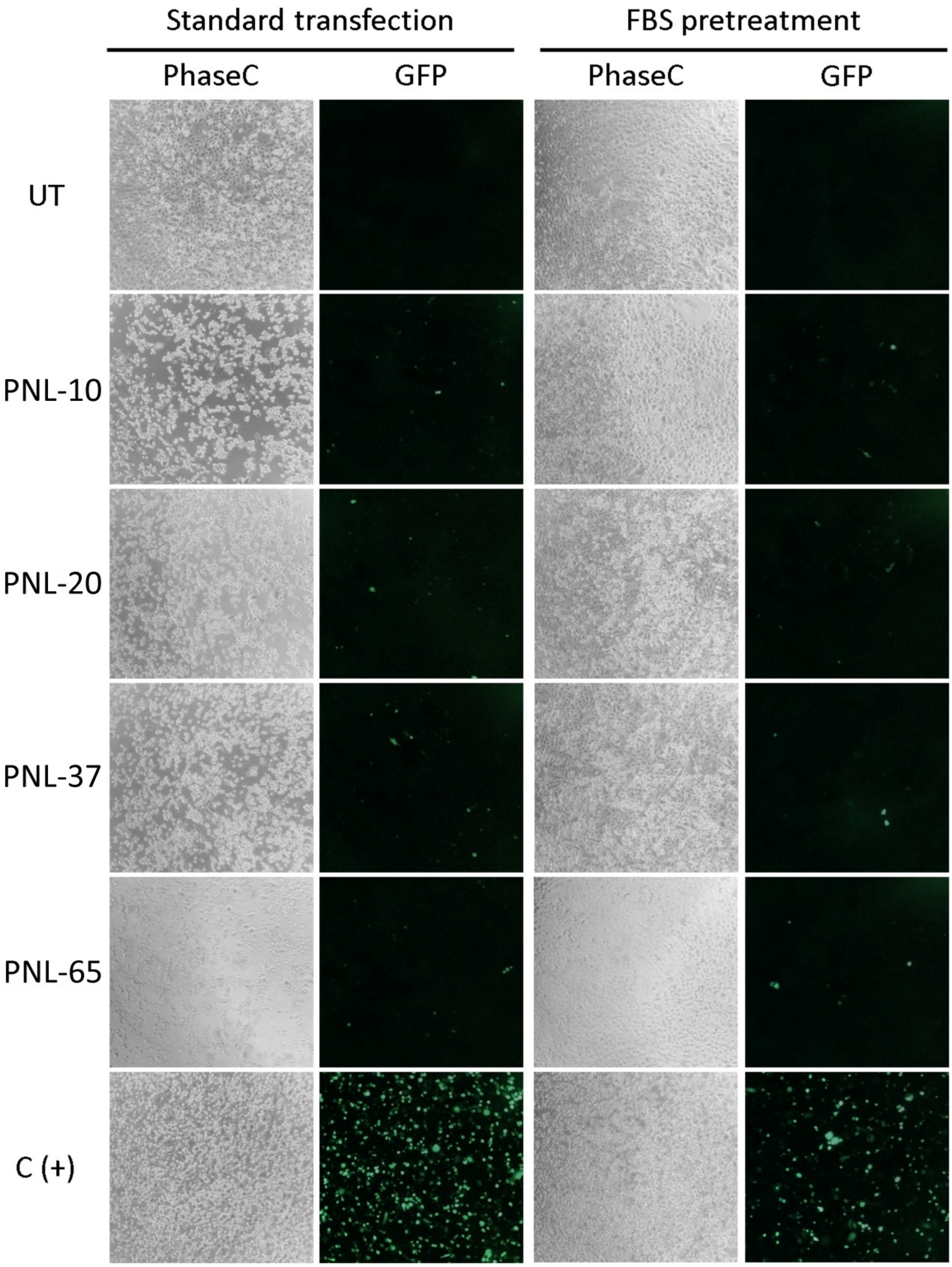

Figure S7. Fluorescence microscopy images of PC3 cells subjected to 72h transfection with the polyplexes.

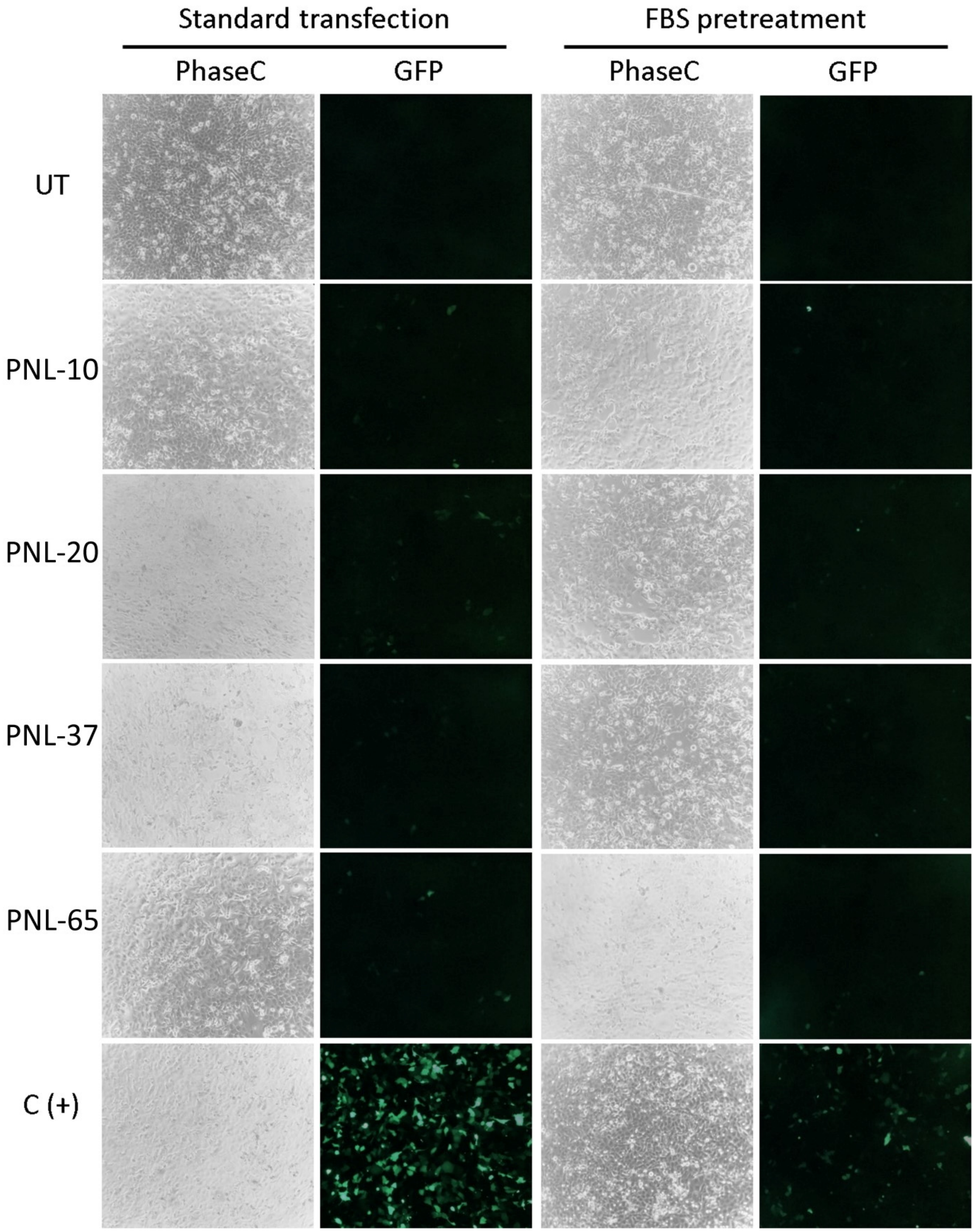

Figure S8. Fluorescence microscopy images of H1299 cells subjected to 72h transfection with the polyplexes.

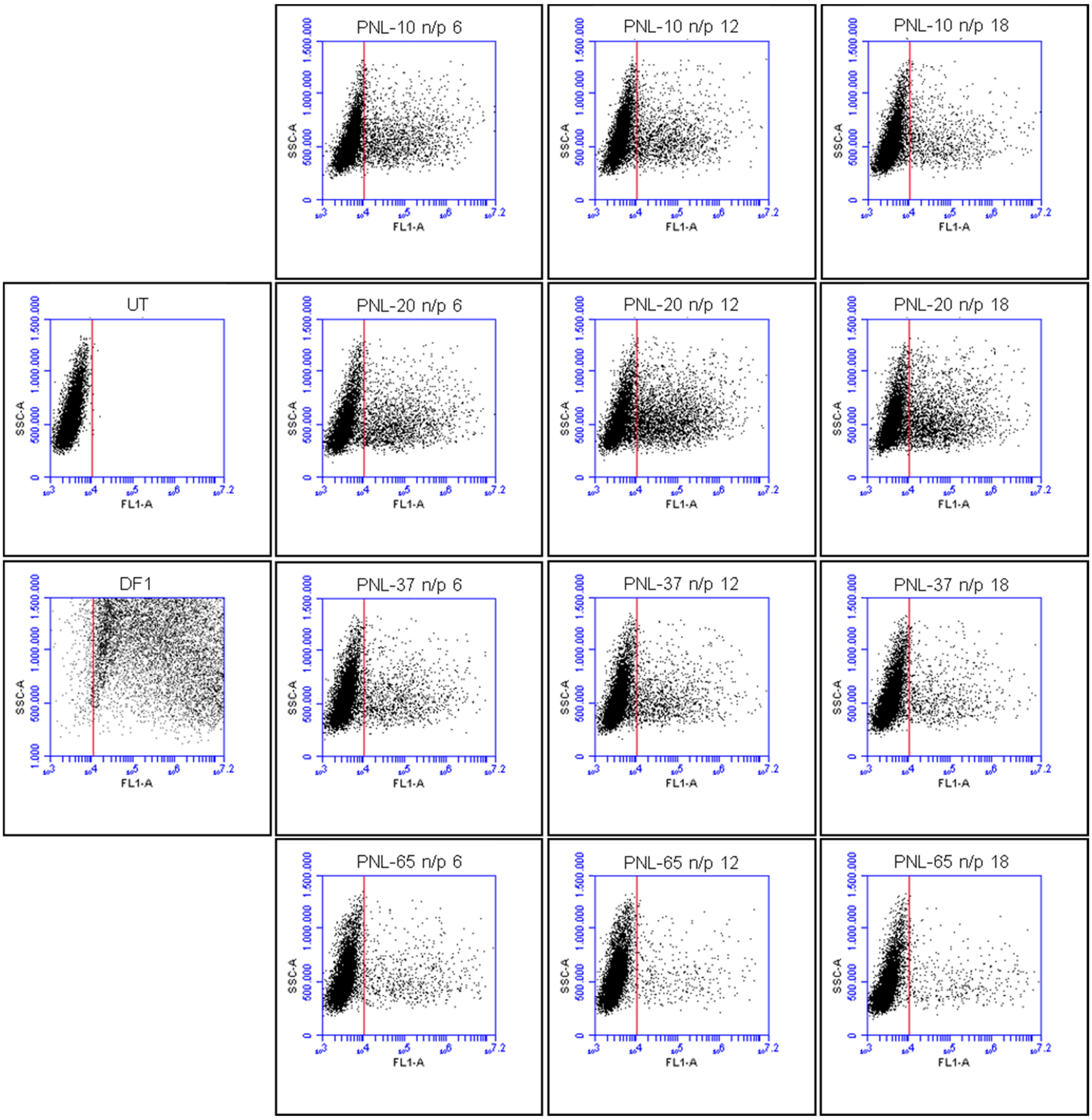

Figure S9. Flow cytometry scatter plots of PC3 cells transfected with polyplexes prepared at three N/P ratios. Cells were treated with the polyplexes for 72h as described under materials and methods and analyzed for GFP signal. UT- untreated cells; DF1- DharmaFECT 1 commercial transfection reagent.

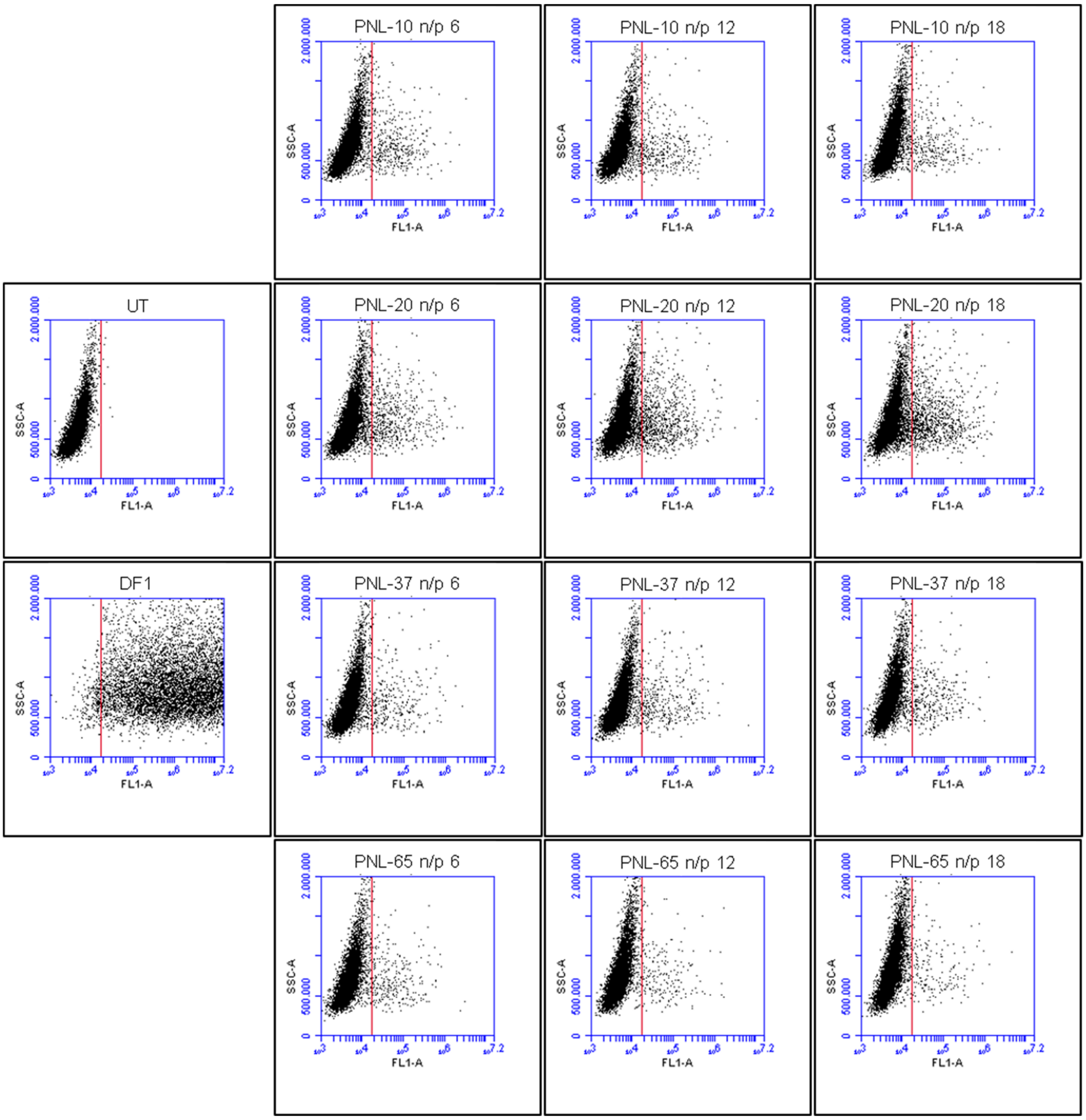

Figure S10. Flow cytometry scatter plots of H1299 cells transfected with polyplexes prepared at three N/P ratios. Cells were treated with the polyplexes for 72h as described under materials and methods and analyzed for GFP signal. UT- untreated cells; DF1- DharmaFECT 1 commercial transfection reagent.
